# Evolution and development of the bird chondrocranium

**DOI:** 10.1186/s12983-021-00406-z

**Published:** 2021-04-29

**Authors:** Evelyn Hüppi, Ingmar Werneburg, Marcelo R. Sánchez-Villagra

**Affiliations:** 1 Universität Zürich, Paläontologisches Institut und Museum, Karl-Schmid-Straße 4, 8006 Zürich, Switzerland; 2 Senckenberg Center for Human Evolution and Palaeoenvironment (HEP) an der Eberhard Karls Universität, Sigwartstraße 10, 72076 Tübingen, Germany; 3 Fachbereich Geowissenschaften der Eberhard-Karls-Universität Tübingen, Hölderlinstraße 12, 72074 Tübingen, Germany

**Keywords:** Heterochrony, Skull, Disparity, Homology, Ontogeny, Aves

## Abstract

**Background:**

Birds exhibit an enormous diversity in adult skull shape (disparity), while their embryonic chondrocrania are considered to be conserved across species. However, there may be chondrocranial features that are diagnostic for bird clades or for Aves as a whole. We synthesized and analyzed information on the sequence of chondrification of 23 elements in ten bird species and five outgroups. Moreover, we critically considered the developmental morphology of the chondrocrania of 21 bird species and examined whether the diversity in adult skull shape is reflected in the development of the embryonic skull, and whether there are group-specific developmental patterns.

**Results:**

We found that chondrocranial morphology is largely uniform in its major features, with some variation in the presence or absence of fenestrae and other parts. In kiwis (*Apteryx*), the unique morphology of the bony skull in the orbito-nasal region is reflected in its chondrocranial anatomy. Finally, differences in morphology and chondrification sequence may distinguish between different Palaeognathae and Neognathae and between the Galloanserae and Neoaves. The sequence of chondrification is largely conserved in birds, but with some variation in most regions. The peri- and prechordal areas in the base of the chondrocranium are largely conserved. In contrast to the outgroups, chondrification in birds starts in the acrochordal cartilage and the basicranial fenestra is formed secondarily. Further differences concern the orbital region, including early chondrification of the pila antotica and the late formation of the planum supraseptale.

**Conclusion:**

Synthesizing information on chondrocranial development confronts terminological issues and a lack of comparable methods used (e.g., different staining; whole-mounts versus histology). These issues were taken into consideration when assessing differences across species. The summary of works on avian chondrocranial development, covered more than a century, and a comparison of the chondrification sequence among birds could be conducted. Future studies could test the hypothesis that chondrocranial disparity in Aves, in terms of the shape and proportion of individual elements, could be as large as adult skull disparity, despite conserved developmental patterns and the richness of forms in other (dermal) portions of the skull.

**Supplementary Information:**

The online version contains supplementary material available at 10.1186/s12983-021-00406-z.

## Background

There is much disparity among birds, as reflected in variation in skull shape [1, 2]. Adult diversity has been intensively studied in recent years, mainly with methods that quantify shape variation, aiming at understanding patterns across groups [3–6], evolutionary patterns of modularity [7], or comparisons with the fossil record [8–10]. The development of the skull, on the other hand, has not been subject of synthetic treatment. In particular, although the chondrocranium has been studied for more than a century, an evaluation of the largely descriptive work of individual stages is lacking. The notable synthesis of de Beer [11] did not benefit from the phylogenetic, methodological, and conceptual framework we now have available, and several studies have been performed since then. Comparative work on other diapsids can also provide new insights into skull evolution in this group [12–17].

Reportedly, only a few chondrocranial features distinguish birds from non-avian sauropsids, in particular from crocodilians [11]. Crocodilians are similar to birds [18, 19] in the arrangement of chondrocranial features and in the general sequence of chondrification [20]. This concordance has been discussed as supporting a closer relationship of birds and crocodilians to each other than to other sauropsids [18, 19, 21]. Within crocodilians, there are commonalities in the chondrocrania of Alligatoridae [22] and Crocodylidae [19, 23] but chondrocranial differences within and between closely related species have also been reported [24].

No specific chondrocranial features to discriminate among the major avian taxa are known; essentially the chondrocrania are considered to be similar [11, 25]. The distinction between Palaeognathae and Neognathae is mainly based on the pterygoid-palate connection in the adult skull [26, 27]. In the embryonic cartilaginous skull, however, Palaeognathae and Neognathae cannot be distinguished from each other [28] as a robust criterion for comparing chondrocrania among species is lacking so far. The recently developed “tempus optimum” approach to define comparable stages in chondrogenesis addresses this issue [24]. One way to characterize and compare the development of the skull across species is to examine the onset of ossification of its individual elements. This has been done for many clades of non-avian amniotes [29–32] and for birds [33–41].

Only few authors have dealt with chondrification sequences of the skull [12, 42, 43]. The latter does not run parallel with the ossification sequence [11]. The chondrification sequence in birds has not been analyzed, although the chondrocranium of many species has been described [44]. This is mainly because of questionable homologization, missing analytical methodology, and a stable within-bird phylogeny. We confront these issues in this paper.

We present a comprehensive analysis on the chondrification sequence and the diversity of the cartilaginous embryonic skull of birds based on detailed descriptions published since the nineteenth century. We compare patterns of chondrification among ten bird species and five outgroup representatives, and we discuss homology and variation in the morphology of their chondrocrania and those of eleven other bird species. We focus on the question if the great diversity of bird skulls is reflected in chondrocranial disparity, and whether the sequence of chondrification of the embryonic skull is preserved among different bird taxa.

## Results

### Chondrocranial characters of birds: morphology, variation and homologization of structures, and sequence of chondrification in the skull

**Fig. 1:**
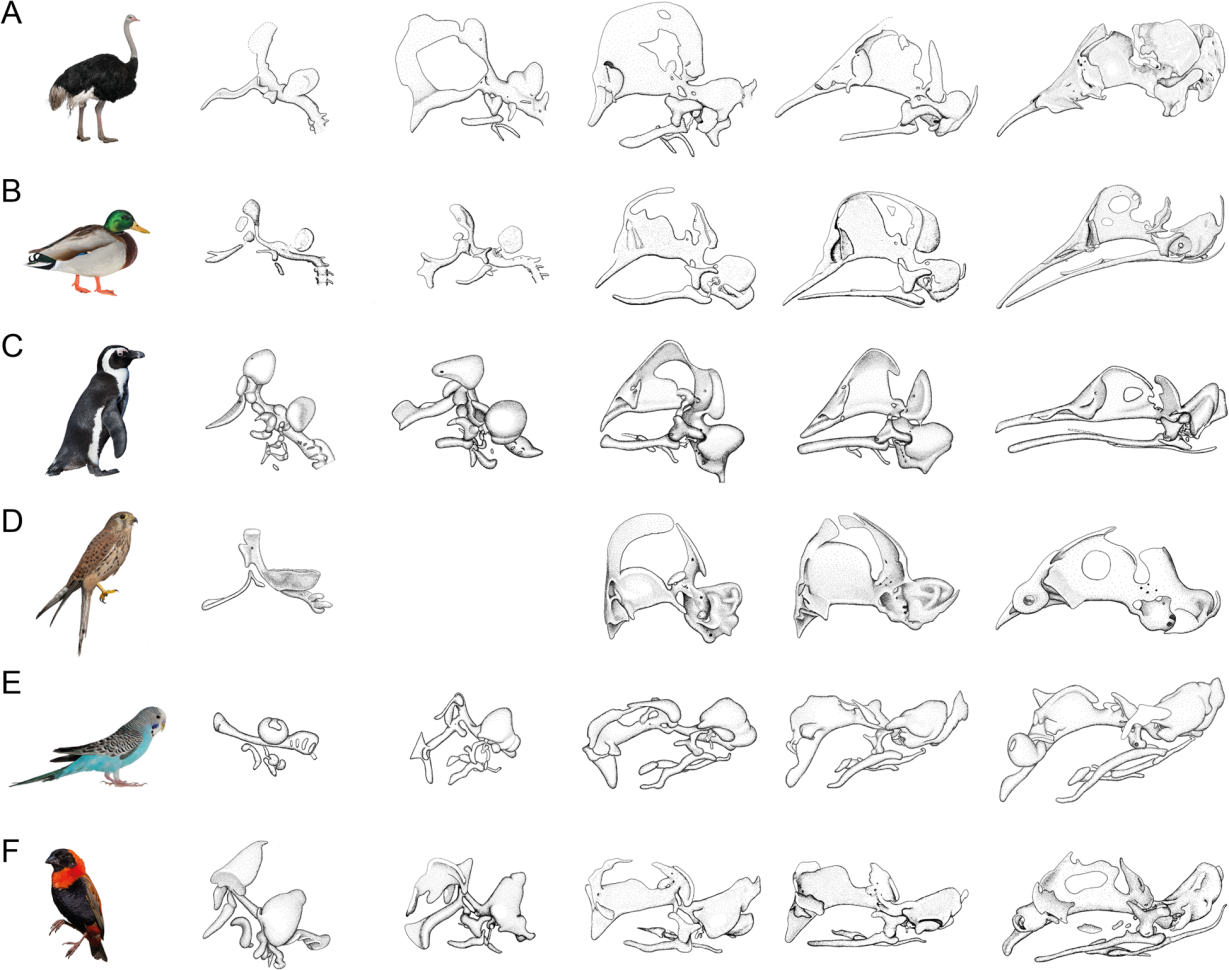
Comparable chondrocranial stages of six birds. Stages increasing in age from left to right. **a** *Struthio* sp. [25, 45], **b** *Anas platyrhynchos* [46], **c** *Spheniscus demersus* [47], **d** *Falco tinnunculus* [48], **e** *Melopsittacus undulates* [49], and **f** *Euplectes orix* [50]. Drawings by Timea Bodogán, modified from cited sources

**Fig. 2:**
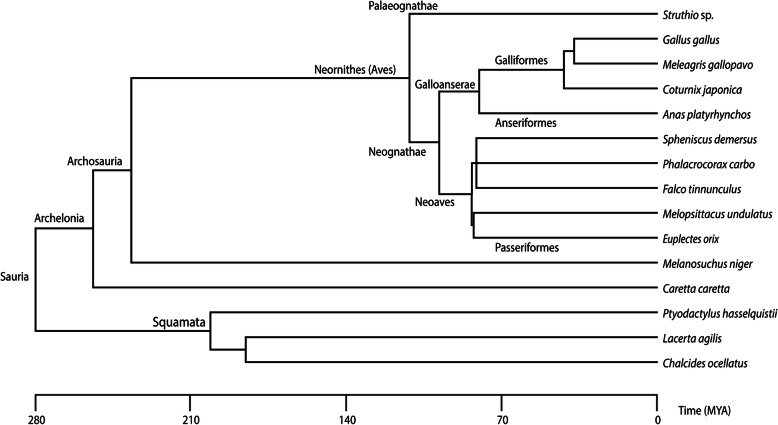
Taxonomic sampling of birds and non-avian sauropsids whose chondrification sequence was compared in this study

**Fig. 3:**
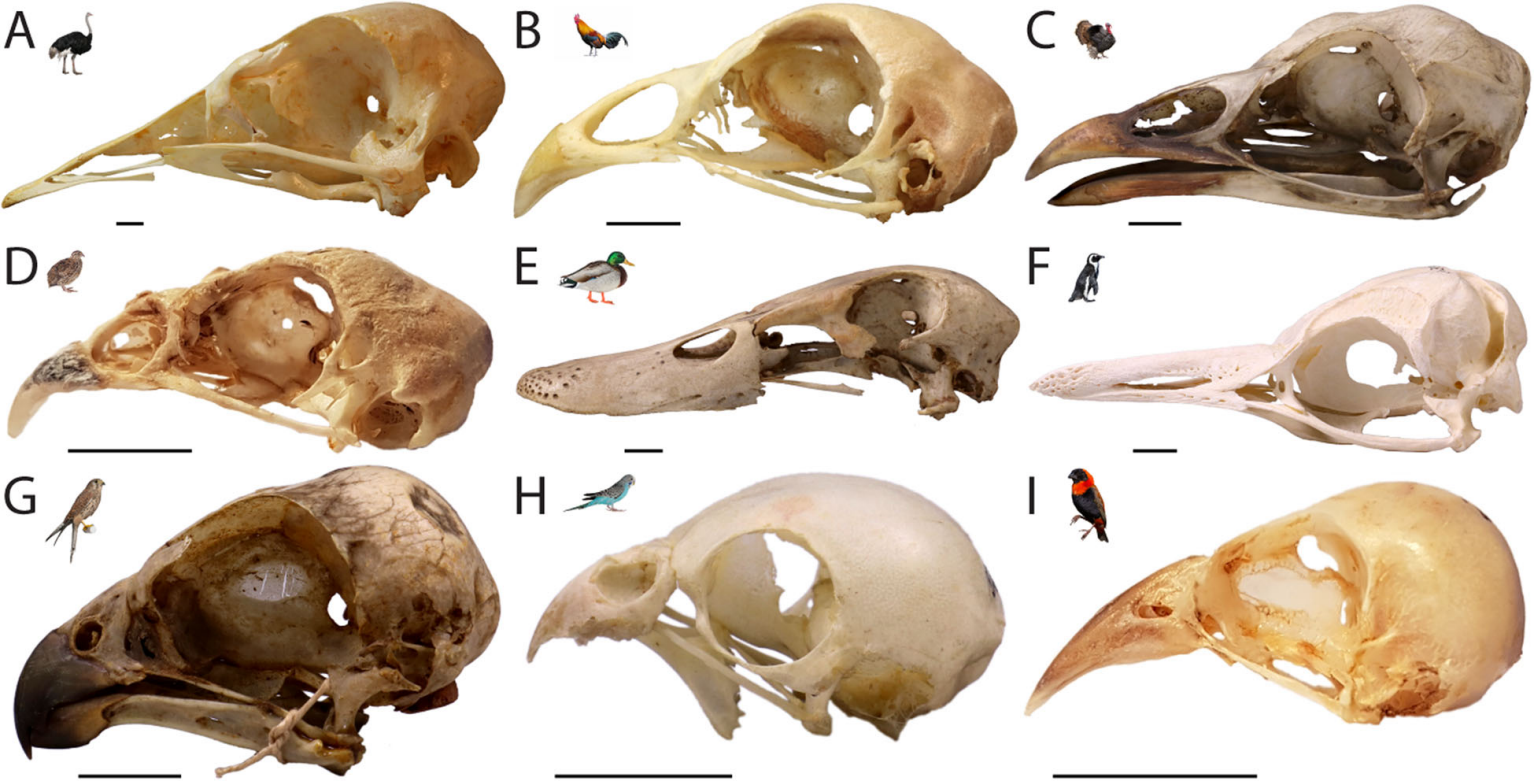
Morphological diversity and disparity in the skull of birds, with examples of taxa considered in chondrocranial studies discussed in this work. **a** *Struthio camelus* (NMB 5516), **b** *Gallus domesticus* (PIMUZ 154), **c** *Meleagris gallopavo* (NMB 881), **d** *Coturnix japonica* (SMF 7180), **e** *Anas platyrhynchos* (NMB C. 833), **f** *Spheniscus demersus* (NMB 5634), **g** *Falco tinnunculus* (NMB 1351), H) *Melopsittacus undulates* (NMB 5898), and I) *Euplectes orix franciscanus* (SMF 2763). Scale bars equal 10 mm

The supposed conservatism of chondrocranial morphology (Fig. 1) in the group [11, 25, 28] (Fig. 2) is in strong contrast to the disparity of adult avian skull shape [2] (Fig. 3). The avian chondrocranium is characterized by only a few specific features that distinguishes it from that of non-avian sauropsids and which are shared with other archosaurs [11]. There are descriptions of chondrocranial development of particular species [25, 28, 45–58] and several aspects of the development of the bird chondrocranium have been discussed before [11, 44, 59, 60], but without a consideration of phylogeny as currently understood.

The onset of the chondrification process in the skull appears in the pre- and perichordal elements of the chondrocranium. The last elements of the chondrocranium that chondrify are the most anterior ones, the cupola anterior and the atrioturbinal. This sequential pattern is characteristic for the chondrocranial development of vertebrates in general [11, 24, 59]. The cormorant *Phalacrocorax carbo* is the only documented exception among birds in which the onset of chondrification was not in the chondrocranium sensu stricto, but the chondrification of Meckel’s cartilage and hyoid apparatus took place before that of the neurocranium [61].

## The basal plate and the fenestra basicranialis posterior

### Process of development

#### Parachordal and acrochordal cartilage

**Fig. 4:**
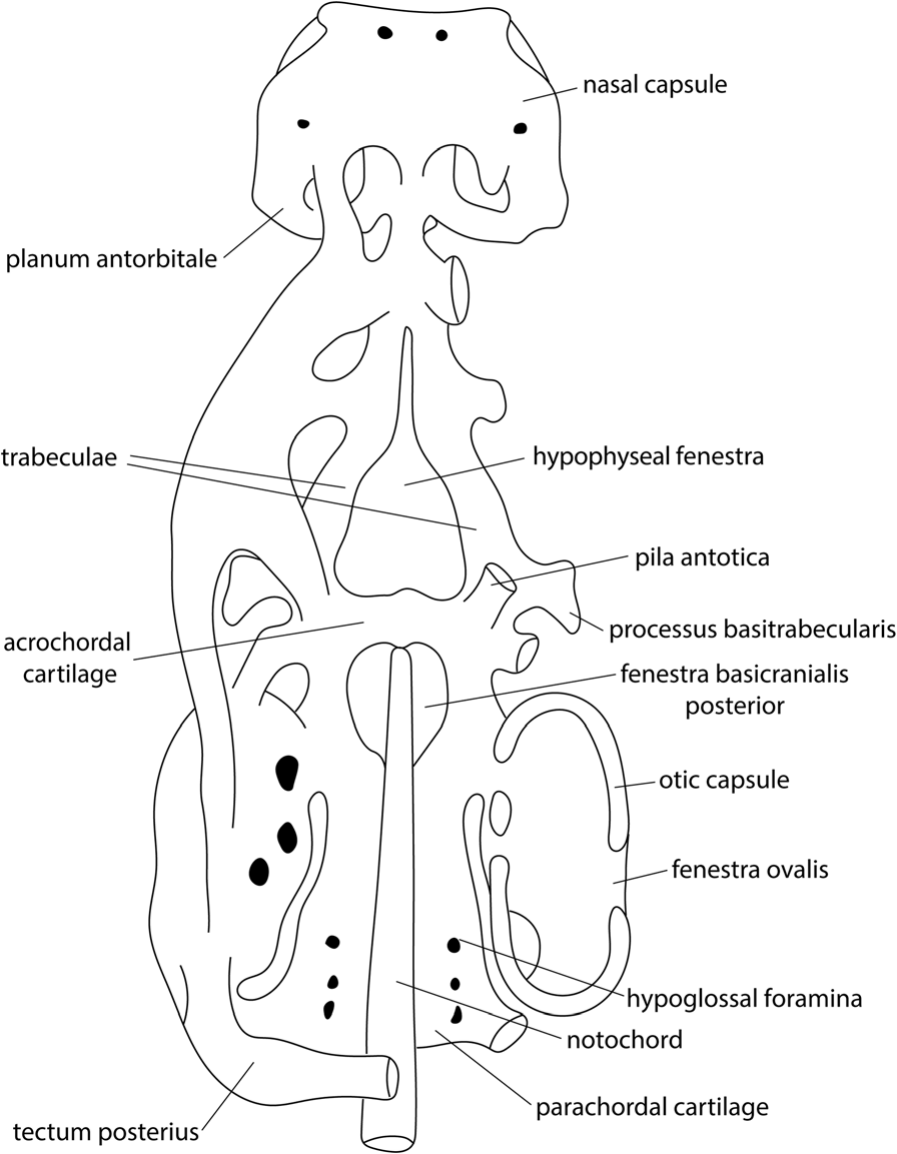
Schematic representation of a dorsal view of an amniote chondrocranium. Redrawn from Goodrich [62] and Mickoleit [63]

The parachordal cartilage (Fig. 4) constitutes the posterior part of the basal plate. Posteriorly, the vertebral column connects to the basal plate [62], while at the anterior edge of the parachordal cartilage is the fenestra basicranialis posterior [11] (Fig. 4). Along its medial axis it encloses the notochord [11, 48] (Fig. 4), in whose posterior region the hypoglossal foramina (Fig. 4) are located on both sides [64]. Together with the anteriorly located acrochordal cartilage (Fig. 4), the parachordal cartilage forms the base of the chondrocranium [64]. In birds, the parachordal cartilage is continuous below and above the notochord from its first chondrification on [11] (Table 1), and is therefore occasionally called perichordal [46, 47, 65]. The parachordal cartilage in the kiwi *Apteryx australis* [57] was interpreted as being paired [44, 47, 64], despite the detailed description of its formation and its fusion in the precartilage state [57]. Suschkin [48] argued that the dye (carmine) used by Parker [57] was not suitable for staining intercellular substance and that because of the difference in chondrogenesis between the median and lateral part of the parachordal cartilage, the median aspect was less obvious and gave the impression of a paired anlage. Sonies [64] also mentioned that a paired impression of the structure is possible, since the median portion of the parachordal cartilage is thinner than the lateral part.

**Table 1:** A selection of chondrocranial features typical for birds listed by de Beer [11]

Distinctive features of birds and crocodilians (de Beer 1937:463):	
“*(i) The presence of a median prenasal process.*“	
“*(ii) The formation of infrapolar processes.*“	
“*(iii) The formation of subcapsular process or metotic cartilages.*“	
Features that birds share with crocodilians and chelonians (de Beer1937:463):	
“*(i) The intraparachordal course of the notochord.*“	
Features shared by reptilians in general (de Beer 1937:463):	
“*(ii) The formation of the supratrabecular bar (except Ophidia).*“	
“*(iii) The disposition of the orbital cartilage (planum supraseptale) and pila antotica.*“	

According to Suschkin [48] the unpaired anlage of the parachordal cartilage in birds is the result of an accelerated and direct development to this condition, in contrast to an originally paired formation and later fusion. Sonies [64] also argued for an accelerated development in the domesticated fowl *Gallus gallus*, where different chondrocranial characters were already continuous during their anlage, whereas in the dabbling duck *Anas platyrhynchos* the same chondrocranial anlagen developed independent from each other.

Suschkin [48] further specified that a paired anlage of the parachordal cartilage may exist in the precartilaginous state, while chondrification takes place in the fused parachordal anlage. A paired parachordal cartilage in the pre-cartilaginous state was only described in *Apteryx* [57], while in all other descriptions of birds the parachordal cartilage in the precartilage state was already a uniform structure. Also, de Beer [11] emphasized that the parachordal cartilage of many birds is strictly speaking not a paired structure.

In other archosaurs, such as the slender-snouted crocodile *Mecistops cataphractus* (“*Crocodylus cataphractus*”) [20] and the saltwater crocodile *Crocodylus porosus* (“*Crocodylus biporcatus*”) [66], the parachordal cartilage is described as a continuous element, whereas in the alligator *Caiman yacare* paired [19] and unpaired [22] cartilaginous states were described.

The acrochordal cartilage (Fig. 4) constitutes the anterior portion of the basal plate between the parachordal cartilage and the trabeculae. The acrochordal cartilage does not lie at the same level as the parachordal cartilage, as it is more dorsally oriented, and separates the fenestra basicranialis posterior and the hypophyseal fenestra [17]. The unpaired structure delimits the fenestra basicranialis posterior anteriorly and forms the posterior border of the hypophyseal fenestra [11].

#### Fenestra basicranialis posterior

The formation of the fenestra basicranialis posterior (Fig. 4) is described as an unchondrified space left between the anterior aspects of the parachordals [62] and the acrochordal cartilage (primary origin) (*Apteryx* [57], *Gallus* [64], *Anas* [46, 64], *Phalacrocorax* [61]), or by resorption of intercellular substance in the anterior part of the parachordal cartilage (secondary origin) (*Gallus* [58], the falcon *Falco tinnunculus* [48], the weaver *Euplectes orix* [50], the pigeon *Columba livia* [67, 68], the dove *Streptopelia senegalensis* [54]). The fenestra of primary origin is filled with diffuse mesenchyme [61], whereas the secondarily formed fenestra is covered with a thin layer of connective tissue [48, 50, 69]. Similarly, in squamates the primary basicranial fenestra is filled with a layer of undifferentiated cells [12, 15]. In birds, not only variation in the formation of the basicranial fenestra among species was described, but also intraspecific variation is documented (*Gallus* [69, 70]).

Sonies [64], who worked mainly with whole-mount staining (Table S1, Additional file 1), observed a primary origin in which the basicranial fenestra was formed by leaving a space free when the cartilages of the basal plate merged whereas authors working with histological sections described a secondary origin [58, 69] in which the basicranial fenestra was formed by resorption of the previously uniform cartilage of the basal plate. Sonies [64] mentioned that the differences in his findings were most likely due to differences in the method used. While in *Gallus* the intraspecific variation must relate to the different methodological approaches, no intraspecific variation was observed in *Anas*. Both studies on *Anas* used whole-mounts [46, 64], but de Beer and Barrington [46] also used histology. It is unclear which method they focused on and for which stages which methods were used.

In *Apteryx* [57] and *Phalacrocorax* [61], where also a primary origin of the basicranial fenestra was described, histological sections were used. The sections of the former were stained with borax-carmine, the ones of the latter with haematoxylin and eosin, both stainings were often used in histology (Table S1, Additional file 1).

In the ostrich *Struthio* sp. [25, 45, 56, 65], the information about the presence of the posterior basicranial fenestra is contradictory (Table 4). In other birds, several of them with relative large eyes, the formation of a basicranial fenestra was not observed (the nightjar *Caprimulgus pectoralis* (“*Nyctisyrigmus pectoralis pectoralis*”) [65], the emu *Dromaius novaehollandiae* (“*Dromaeus novae hollandiae*”), the large ratite *Rhea americana* [56]), or not mentioned.

**Fig. 5:**
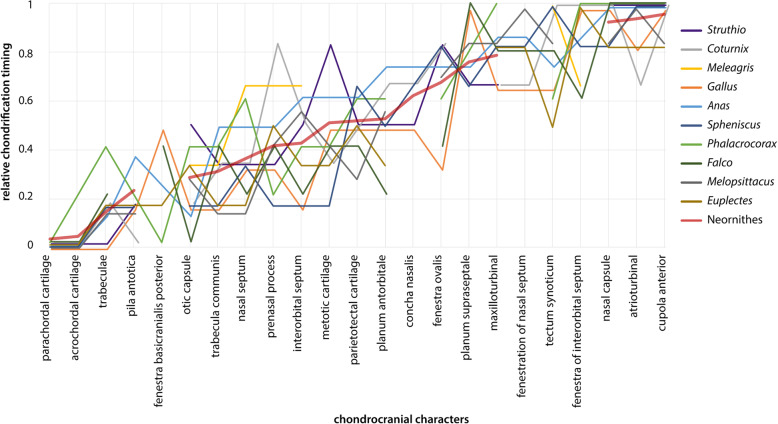
Chondrification sequence of 23 chondrocranial elements in all ten birds and their reconstructed consensus line (Neornithes). The chondrocranial characters are ordered chronologically for Neornithes. The Y-axis shows the relative timing of chondrification in which “0” represents the appearance of the first cartilage in the chondrocranium and “1” the appearance of the last one. Some chondrification events have not been described for certain species, resulting in gaps in the sequences shown

In the first description of *Struthio* [56] in the literature, a stage where dermal ossifications are already present, a basicranial fenestra was not described. Brock [25] did not describe a basicranial fenestra in *Struthio* either, but labelled it in a figure of a relative early embryo (p. 229: text-Fig. 5). Based on Brock’s [25] work, de Beer [11] mentioned a basicranial fenestra for *Struthio*. Frank [65] worked with a more extensive series of *Struthio* embryos, but could not find evidence for the fenestra. Lang [45] mentioned the absence of the basicranial fenestra for a late stage *Struthio* because of its fusion with the hypophyseal fenestra due to the atrophied acrochordal cartilage.

A similar continuity between the two fenestrae was described for the sparrow *Passer* sp. and the starling *Sturnus* sp. [64] and in late stages of *Phalacrocorax* [61]. Gaupp [59] also mentioned the possibility of the union of the two fenestra. The resorption of the median portion of the acrochordal cartilage is variously pronounced in different bird species [64]. In *Streptopelia* [55], the acrochordal cartilage was reduced to a thin strip of cartilage, but the basicranial fenestra remained separate from the hypophyseal fenestra.

In crocodilians, the absence of the fenestra basicranialis posterior was mentioned for several species ([11, 23, 59, 71], *Mecistops* [20], *Caiman* [19, 22]). However, in *Crocodylus* [66], a vacuity formed by regression of the cartilage at the anterior end of the notochord in late ontogeny was described, and Shiino [66] specified that it is the same condition as in late stages in birds. Further, in the black caiman *Melanosuchus niger* too, a “*barely evident*” basicranial fenestra was reported [72] (p. 11).

### Chondrification sequence

#### Perichordal region

The early onset of chondrification around the notochord in the base of the skull was almost the same in all birds (Fig. 5, Table 2). Nevertheless, there is some variation in the sequence of chondrification. Either the acrochordal cartilage (Fig. 5) chondrified slightly earlier than the parachordal cartilage (*Apteryx* [57], *Gallus* [64, 69], *Anas* [46, 64], the quail *Coturnix japonica* [75], *Columba* [68]) (Fig. 5), or chondrification of the parachordal cartilage was more advanced than in the acrochordal cartilage (*Phalacrocorax* [61], *Struthio* [65]).

**Table 2:** Sequence of chondrification in the chondrocranium of birds and non-avian sauropsids. Chondrocranial characters ordered by anatomical regions. For the sequential stages as originally reported see Table S4, Additional file 4 and Table S5, Additional file 5

Species	*Struthio* sp. [25, 45, 56, 65]	*Gallus gallus* [21, 58, 60, 64, 69, 73, 74]	*Meleagris gallopavo* [33, 37]	*Coturnix japonica* [34, 75]	*Anas platyrhynchos* [46, 64]	*Spheniscus demersus* [47]	*Phalacrocorax carbo* [61]	*Falco tinnunculus* [48]	*Melopsittacus undulatus* [49, 51]	*Euplectes orix* [50]	*Melanosuchus niger* [72]	*Caretta caretta* [76]	*Ptyodactylus hasselquistii* [77, 78]	*Lacerta agilis* [15]	*Chalcides ocellatus* [79, 80]
Characters															
acrochordal cartilage	1	1	?	1	1	1	2	1	1	1	2	1	1	1	3
parachordal cartilage	1	1	1	1	1	1	1	1	1	1	4	1	1	1	1
fenestra basicranialis posterior	?	4	?	?	3	–	1	3	–	2	?	3	3	2	3
trabeculae	1	1	2	2	2	2	3	2	2	2	1	1	1	2	1
trabecula communis	3	2	2	3	5	2	3	3	2	2	3	2	3	2	2
otic capsule	4	2	2	2	2	2	3	1	3	3	4	1	2	3	2
fenestra ovalis	6	3	?	6	7	6	4	3	6	4	?	?	4	7	6
metotic cartilage	6	4	?	3	6	2	3	3	4	3	?	–	–	–	–
nasal septum	3	3	3	3	5	3	4	2	2	2	3	3	4	4	3
fenestration of nasal septum	–	5	?	5	8	6	?	5	8	6	–	?	8	10	6
prenasal process	3	3	3	6	5	2	2	3	4	4	?	–	–	–	–
planum antorbitale	4	4	?	5	7	4	4	2	5	3	4	?	5	5	5
parietotectal cartilage	4	4	?	4	6	5	4	3	3	4	4	3	5	7	4
nasal capsule	7	7	?	7	9	6	6	6	7	6	4	?	6	9	5
cupola anterior	7	7	?	7	9	7	6	6	7	6	–	?	6	10	5
concha nasalis	4	4	?	5	7	5	?	?	–	–	4	?	7	?	5
maxilloturbinal	5	5	?	5	8	6	6	5	7	6	?	?	?	?	?
atrioturbinal	7	6	?	5	9	7	6	6	8	6	?	?	?	?	?
interorbital septum	4	2	3	4	6	2	3	2	5	3	4	2	4	2	3
fenestration of interorbital septum	–	7	3	7	8	6	6	4	–	7	?	?	–	8	6
planum supraseptale	5	7	?	?	7	5	5	6	7	?	4	3	4	2	3
pila antotica	2	2	?	1	4	2	2	?	2	2	4	4	4	2	5
tectum synoticum	7	5	4	7	7	7	4	5	7	4	5	5	7	6	5

In *Struthio* [65], both structures appeared in the same stage. Frank [65] mentioned for the parachordal cartilage only its presence without describing the cartilaginous state, whereas the acrochordal cartilage was described as a faint mesenchymal condensation.

In *Phalacrocorax* [61], the posterior portion of the parachordal cartilage contained some intercellular substance, while the acrochordal cartilage was made of condensed mesenchyme. The staining they used was the same, and it was used also in other studies [50, 69], so the method cannot be the reason for the reported differences. The characteristic pattern of birds with the onset of chondrification in the acrochordal cartilage [69] is not reflected in the chondrification sequence of the birds considered in this study, since in most birds, the acrochordal and parachordal cartilage appeared in the same stage.

Similar to birds, the parachordal cartilage in crocodilians was among the first chondrocranial parts to appear (*Mecistops* [20, 23]). Only in *Melanosuchus* [72] the onset of chondrification was described in the prechordal region in the trabeculae.

#### Fenestra basicranialis posterior

**Fig. 6:**
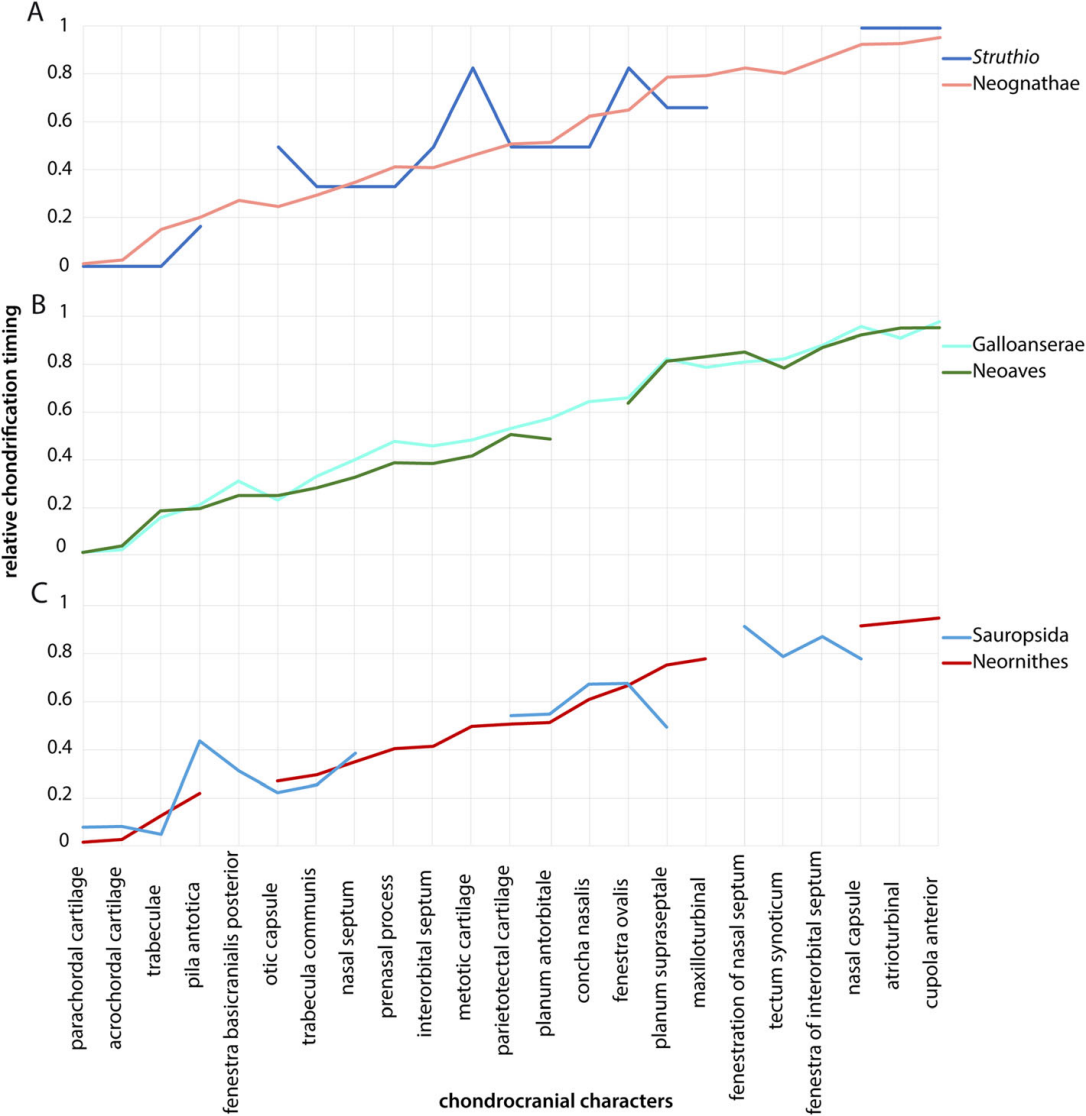
Comparison of the chondrification sequence between different groups. The chondrocranial characters on the X-axis are ordered chronologically for Neornithes. The Y-axis shows the relative timing of chondrification in which “0” represents the appearance of the first cartilage in the chondrocranium and “1” the appearance of the last one. Some chondrification events have not been described for certain species, resulting in gaps in the sequences shown. **a** *Struthio* as representative of the Palaeognathae compared to the Neognathae, **b** comparison between Galloanserae and Neoaves, and **c** the ancestral chondrification sequence (Sauropsida, consensus line of all reptiles in this study (Table 2) compared to Neornithes

The formation of the fenestra basicranialis posterior (Fig. 4) follows the formation the basal plate and the onset of chondrification in the trabeculae (Fig. 5, Table 2). Only in *Phalacrocorax* [61], the basicranial fenestra formed before the trabeculae and much earlier compared to the formation in the other bird (Fig. 5). In the total sequence of chondrification, the primary origin of the fenestra (i.e., formed by surrounding cartilage) in *Anas* [46, 64] is not reflected in the chondrification sequence by an earlier appearance compared to a fenestra of secondary origin (i.e., formed by degeneration). In Neoaves, the basicranial fenestra formed slightly earlier than in Galloanserae (Fig. 6b).

## The prechordal region

### Process of development

#### Trabeculae and trabecula communis

**Fig. 7:**
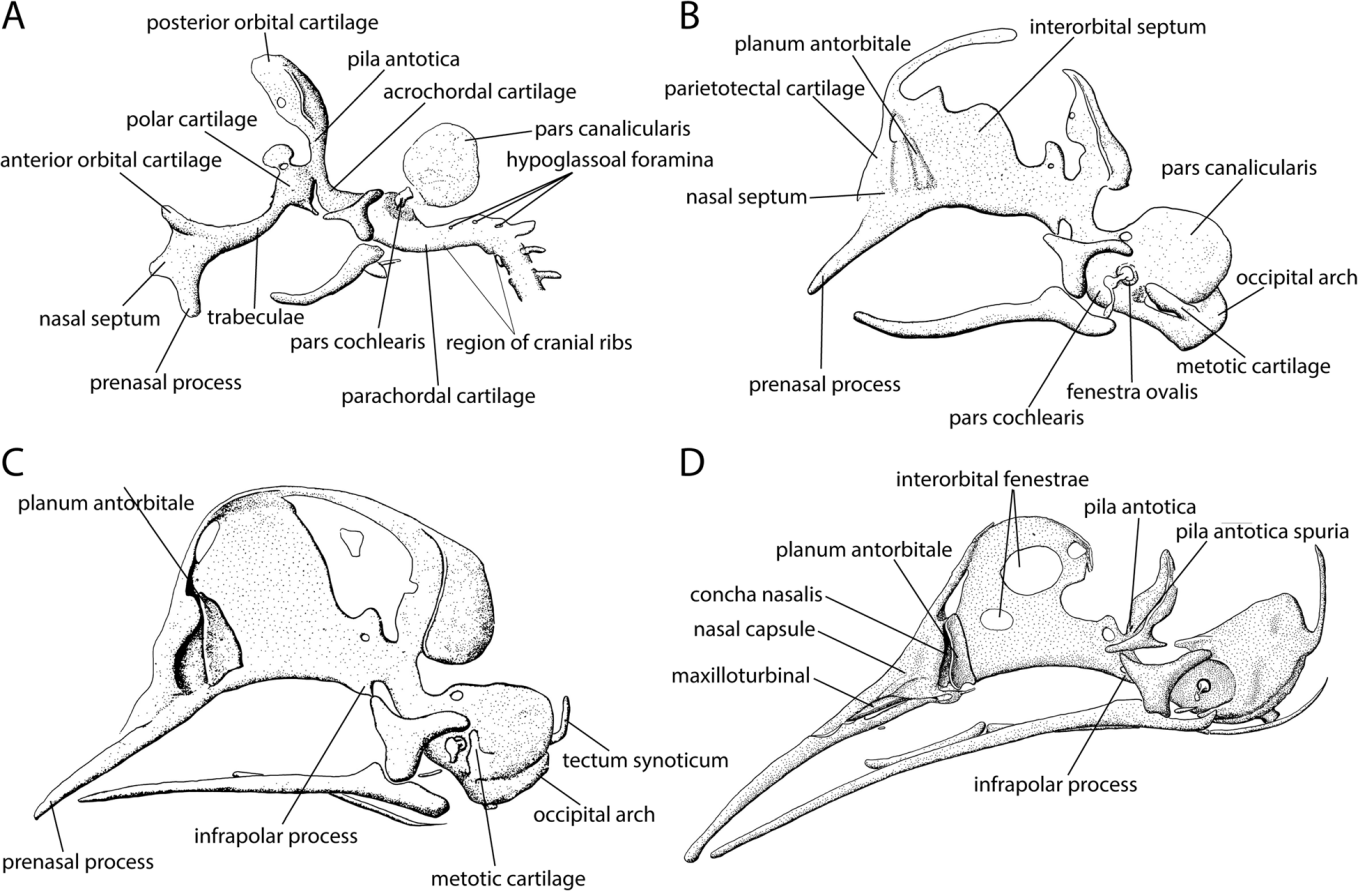
Development of the prechordal region in *Anas platyrhynchos* [46]. Lateral view of chondrocranial stages: **a** stage 8 (7.5 days), **b** stage 10 (9 days), **c** stage 11 (9.5 days), and **d** stage 13 (14 days). Drawings by Timea Bodogán, modified from cited source

The paired trabeculae (Figs. 4 and 7a) are situated angled in front of the basal plate and rostral to the notochord. Between the trabeculae and the basal plate is the polar cartilage (Fig. 7a). The trabeculae are the first chondrocranial elements that appear in the prechordal region [81]. In some birds, the trabeculae were continuous with the polar cartilage from their first appearance (*Falco* [48], the penguin *Spheniscus demersus* [47], *Gallus* [21, 51], *Struthio* [25, 65], *Euplectes* [50], the budgerigar *Melopsittacus undulatus* [49]). In others, the two elements were, although continuous, still distinguishable from each other (*Gallus* [64, 69], *Phalacrocorax* [61]) or chondrified as independent elements (*Sturnus*, *Anas* [64], *Coturnix* [75]).

**Fig. 8:**
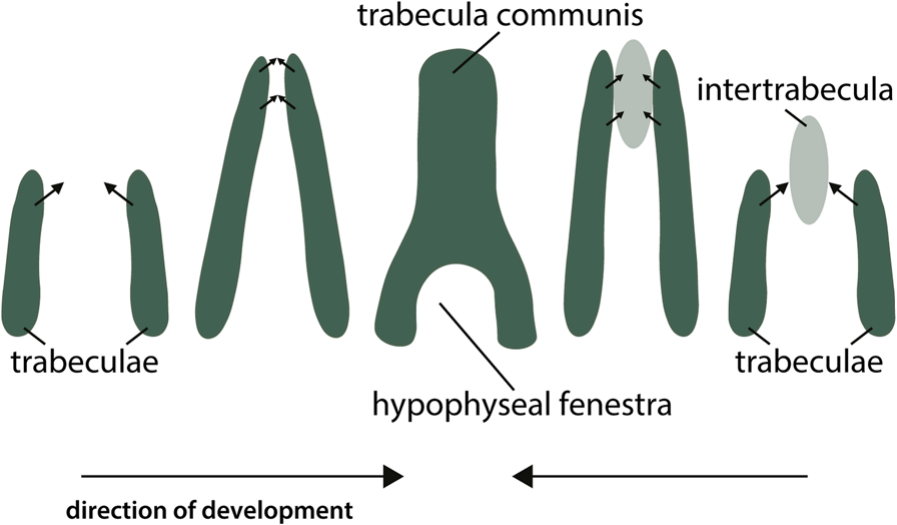
Formation of the trabecula communis. In most birds, the paired trabeculae have been described to grow rostrally together in the midline (left) to form the trabecula communis (middle), whereas in *Falco* and *Coturnix* the trabeculae fused with an intertrabecula (right) to form the trabecula communis. Modified from Wada et al. [82]

Between the caudal portions of the trabeculae lies the hypophyseal fenestra (Figs. 4 and 8). At their rostral ends, the trabeculae merge continuously into the trabecula communis (Fig. 8). Two types of formation of the trabecula communis are described in the literature: either by joining each other at the rostral end (*Gallus* [58, 64], *Anas* [46, 64], *Phalacrocorax* [61], *Spheniscus* [47], *Euplectes* [50], *Melopsittacus* [49]) (Fig. 8), or through fusion with a median element, the intertrabecula (*Falco* [48], *Coturnix* [75]) (Fig. 8). In *Columba*, the trabeculae were continuous at their anterior ends before the intertrabecula appeared [67]*.*

#### Intertrabecula

The intertrabecula (Fig. 8) has been described as a median structure located between the trabeculae and anterior to the hypophyseal fenestra [21]. It is controversial whether it is an independent cartilaginous element or only a histological differentiation in the chondrification process of the prechordal region. An intertrabecula in birds was first briefly mentioned for the hoatzin *Opisthocomus hoazin* (“*Opisthocomus cristatus”*) [83] and *Gallus* [84], followed by the observation of the structure in other birds (*Falco* [48], *Columba* [67, 68], *Dromaius* [45], *Euplectes*, *Gallus*, *Passer* [21], *Coturnix* [75], *Streptopelia* [54]). The nature of the intertrabecula is subject of conflicting reports [11]. Most authors described or figured mesenchymal condensations in the intertrabecular region [21, 48, 65, 67, 69] in close relation to the trabeculae [67, 69]. However, it has been questioned that slight histological differences between the two structures [11, 47, 49, 64, 69, 74] justify the definition as an independent element [21, 48]. Histological differences between the trabeculae and the tissue in between have been described in many species, but most authors did not recognise this tissue in birds as an independent element.

In *Euplectes* and *Gallus*, the development of the intertrabecular region is ambiguous. Bellairs [21], in contrast to Engelbrecht [50] and Vorster [69], was able to determine an intertrabecula. Further criticism concerned the structures labelled as intertrabecula [49]. Depending on the description, the base of the nasal septum (*Streptopelia* [53] (p. 218 Fig. 8)), the interorbital septum (*Coturnix* [75], *Dromaius* [45](p. 189 Abb. 12a)), or the prenasal process (*Gallus* [84] (Pl. XXII)) were designated as intertrabecula. In *Opisthocomus* [83], the intertrabecula was mentioned in a late stage shortly before hatching, namely a stage, in which the intertrabecula is normally no longer distinguishable.

Only recently, an intertrabecula with a high degree of chondrification was briefly mentioned for *Coturnix* [75], and a developmental study revealed that the trabeculae and the intertrabecula are developmentally two distinct elements in *Gallus*; the trabeculae originate from postoptic cells, and the intertrabecula from preoptic cells [75]. The occurrence of a chondrified intertrabecula in *Coturnix* [75] is probably due to the designation of the term “intertrabecular bar” for the interorbital septum, which grows upwards.

First mentioned for the marine turtle *Chelonia mydas* (“*Chelone viridis*”) as a mesenchymal element [85], the intertrabecula was reported in some crocodilians too (the American alligator *Alligator mississippiensis* [86, 87], *Crocodylus* [21], *Caiman* [19]).

### Chondrification sequence

#### Trabecular region

Following the cartilages of the basal plate, the trabeculae chondrified next (Fig. 5). First signs of chondrification in *Gallus* [58, 64, 69, 74] and *Struthio* [25, 45, 65] appeared almost synchronously with the cartilages of the basal plate. Parker [58] and Vorster [69] described the chondrification in the prechordal region of *Gallus* at the same stage as when the chondrification took place around the notochord (Fig. 4). Both discriminated between the cartilaginous states of the pre- and postchordal cartilages by pointing out that the former were the younger structures. In *Struthio* [65], the appearance of trabeculae was described as simultaneous with the acrochordal cartilage (Fig. 7a) following the parachordal cartilage (Fig. 7a).

In most non-avian sauropsids [23], the basal plate is the first part to chondrify (*Mecistops* [20]) (Table 2), followed shortly by the trabeculae. In contrast, for *Melanosuchus* it was reported that the trabeculae chondrified before the elements of the basal plate [72], which is also true for the ancestral sequence of bird chondrification (Fig. 6c).

#### Trabecula communis

**Fig. 9:**
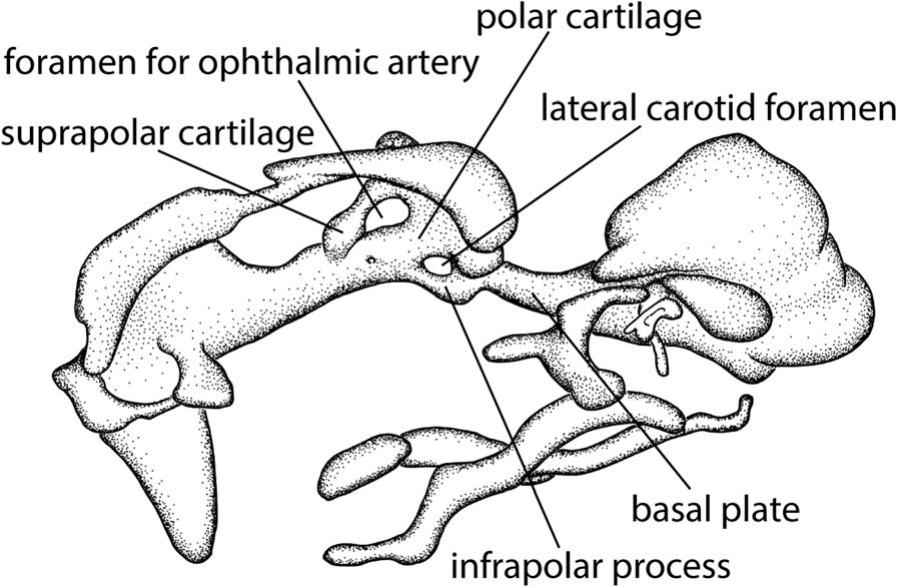
Processes of the polar cartilage in a lateral view of *Melopsittacus undulatus* ([49], stage 4 mirrored). Drawings by Timea Bodogán, modified from cited source

Independent of the described mode of formation, the trabecula communis (Figs. 8 and 9) chondrified subsequent to the trabeculae (Table 2). In Galloanserae, the trabecula communis was formed slightly later than in Neoaves (Fig. 5b).

## The processes of the trabecular-polar region

### Process of development

The prechordal and perichordal regions of the chondrocranium are connected between the trabeculae and the basal plate by several processes (Fig. 9). Parker [58] was the first who mentioned a structure that grows out from the posterior end of the trabeculae as “lingulae sphenoidales”, followed by Parker [57], who called it “basipterygoid process”. Suschkin [48] and Sonies [64] provided a more detailed description of the posterior portion of the trabeculae (trabecular-polar region). Both described one process originating from the posterior end of the trabecular-polar bar, but named it differently, i.e., “processus basitrabecularis” [48] and “processus infrapolaris” [64] (Table S2, Additional file 2). Suschkin [48] homologised the process mentioned by Parker [58] and Parker [57] with his basitrabecular process. Later, Suschkin’s [48] basitrabecular process was labelled as the infrapolar process by Sonies [64]. The different names for the same process refer to its origin, namely the posterior end of trabecular-polar bar. Suschkin [48], however, did not recognize the polar cartilage at the posterior end of the trabeculae and therefore spoke of the process at the base of the trabeculae. Only Sonies [64] identified the polar cartilage continuous with the trabeculae. De Beer and Barrington [46] were the first who noticed the occurrence of two additional processes in this region.

The infrapolar process (Figs. 7c, d and 9) originates at the ventral edge of the polar cartilage and connects the trabeculae with the basal plate ([46, 64] (Fig. 9), whereas the basitrabecular process (Fig. 9) is a lateral projection of the polar cartilage that gets in contact with the palatoquadrate [11, 25, 46, 88, 89].

#### Infrapolar process

The infrapolar process (Figs. 7d and 9), which fuses with the basal plate to border the lateral carotid foramen [11, 62] (Fig. 9), was first described by Sonies [64]. The infrapolar process was in most described birds connected to the ventral surface of the basal plate (*Falco* [48], *Sturnus*, *Passer* [64], *Anas* [46], *Apteryx* [11], *Phalacrocorax* [61], *Melopsittacus* [49, 51]). In certain birds, no contact to the basal plate was established in the described stages (*Gallus* [51, 58, 64, 69], *Apteryx* [57], *Anas* [64], the turkey *Meleagris gallopavo* [37]). Based on the work of W. K. Parker [56] and T. J. Parker [57, 90, 91] on *Struthio* and *Apteryx*, respectively*,* de Beer [11] reported for both taxa an infrapolar process that originated from the basitrabecular process and fused with the ventral surface of the basal plate. The infrapolar process as an outgrowth of the basitrabecular process was also described in *Phalacrocorax* [61] and *Passer* [11]. In *Euplectes*, Engelbrecht [50] found that a homologous process is absent due to the difference in the process of formation and named the structure that bordered the lateral carotid foramen “infracarotid commissure”. The situation in *Spheniscus* [47] was described as being more complicated.

Brock [25] mentioned for an early penguin stage (unknown species) a blunt infrapolar process, and Crompton [47] described in his first stage of *Spheniscus* a short blunt process of the polar cartilage too, but he stated (p. 10): “( …) *although it appears on lateral view to simulate an infrapolar process,* [it] *must not be confused with it*” . In a later stage of *Spheniscus*, the lateral carotid foramen was formed without the contribution of the infrapolar process. Instead, the posterior part of the trabecular-polar bar formed together with the basitrabecular process and the acrochordal cartilage a “infrapolar commissure” that bordered the lateral carotid foramen [47].

Already Sonies [64] mentioned that the connection of the polar cartilage with the basal plate is not constant in birds. It was assumed that the manifestation of the infrapolar process is related to the degree of cranial flexure, which was described to be more pronounced in *Struthio* and *Spheniscus*, while the process was less developed compared to other birds [25, 47, 65]. Likewise, a relation to the size of the eyes was mentioned [25], which are relatively large in flightless birds, such as in ostriches and penguins [92].

The infrapolar process is a feature shared with crocodilians (*Crocodylus* [66], *Alligator* [86], *Mecistops* [20], *Caiman* [19, 22]) (Table 1). For the crocodile, de Beer and Barrington [46] mentioned that the infrapolar process and the basal plate do not connect.

#### Basitrabecular process

**Fig. 10:**
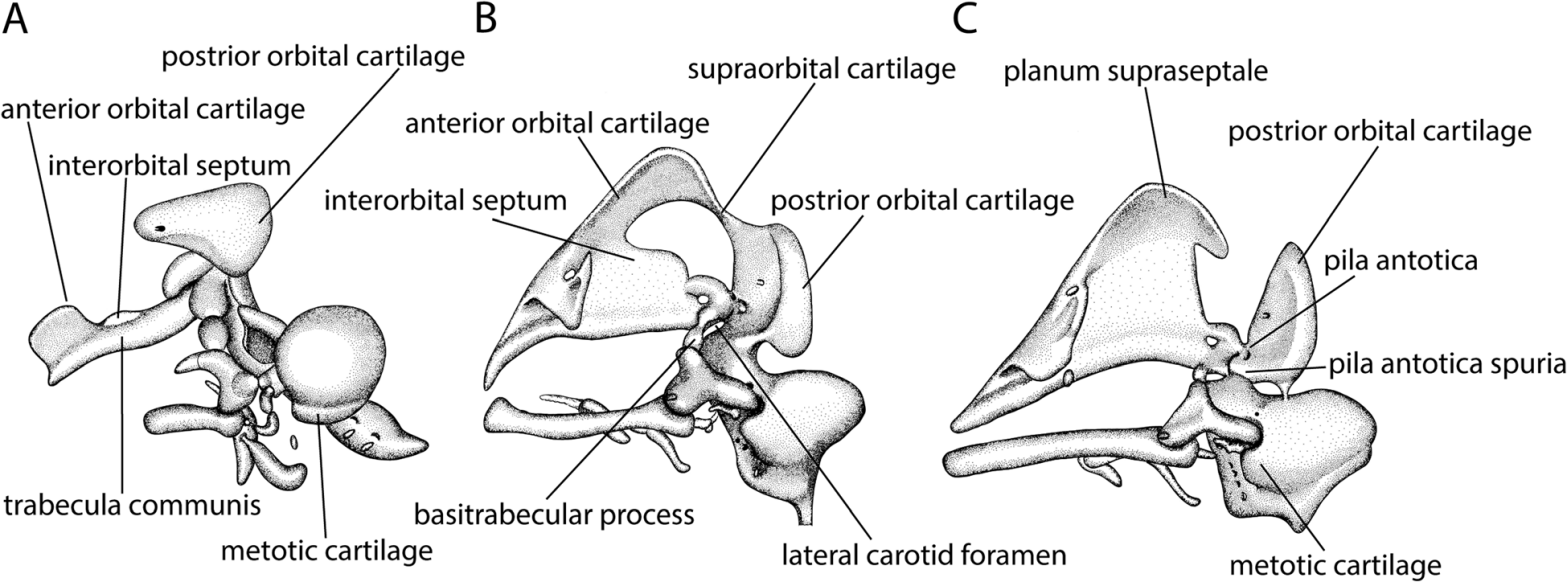
Formation and regression of the anterior part of the orbital cartilage in a lateral view of *Spheniscus demersus* ([47], stage 2, 5 and 6, respectively). Drawings by Timea Bodogán, modified from cited source

In tetrapods, the trabecular-polar bar is transitorily interconnected with the palatoquadrate by the basitrabecular process [11, 88, 93] (Fig. 10b). In birds, only for *Melopsittacus*, the basitrabecular process was not mentioned [49, 51]. Filatoff [67] described the connection between trabecular-polar bar and palatoquadrate in *Columba*, but named it “Columella”. The basitrabecular process was later described in several birds under different names (Table S2, Additional file 2), and in many reported cases only mentioned as “mesenchymal” [69] or “procartilaginous” structure (*Streptopelia* [54], *Anas* [46]). In *Spheniscus* [47] and *Struthio* [65], an independent origin and the chondrification of the process was mentioned, but in most birds, the process developed in continuity with the trabecular-polar bar. An indirect connection of the basitrabecular process with the basal plate was described in *Phalacrocorax* [61], *Columba* [67], *Spheniscus* [47], and *Euplectes* [50]. In *Phalacrocorax* [61], the basitrabecular process lost its contact to the quadrate after the infrapolar process grew out of it and connected to the basal plate. Similar, Filatoff [67] described in *Columba* a process that connected the polar cartilage with the quadrate, but after the break down of the connection, the “trabecular part” of the process took place in the formation of the lateral carotid foramen. A connection to the basal plate was not explicitly mentioned. Lang [51] mentioned for *Columba* a “Basitrabekel”, which she homologized later with the basitrabecular process of *Struthio* “Proc. pterygoideus basisphenoidei” [45], although a connection to the quadrate was missing and the fusion to the basal plate was described. In *Spheniscus* [47], the basitrabecular process was in contact with the polar cartilage, with the palatoquadrate and additionally fused to the acrochordal cartilage.

In *Euplectes*, Engelbrecht [50] described the transitory connection between the quadrate and the polar cartilage as “quadratopolar commissure”. The dorsal part of this commissure was chondrified as “processus basipterygoideus” and contributed to the anterior portion of the “infracarotid commissure”. The commissure ultimately fused with a protrusion of the basal plate to border the lateral carotid foramen. Already Brock [25] stated that the basitrabecular process seems to be a variable structure (p. 235): “*sometimes* ( …) *being reduced or only transitory and projecting as a lateral process from the infrapolar process; in other cases* ( …) *persisting conspicuously throughout development and appearing as a lateral projection of the polar cartilage independent of the infrapolar process* ( …). ”. Likewise, variation in the development of the basitrabecular process is known from lacertids [15, 93]. The lack of clarity concerning the homology of the processes of the trabecular-polar region and thus the use of terms (Table S2, Additional file 2), makes it difficult to follow the development. In addition, the method used and the selection of stages may influence the observed formation process. Sonies [64], who worked mainly with whole-mount staining, observed no basitrabecular process in *Anas*, in contrast to de Beer and Barrington [46], who documented a procartilaginous process in certain early stages.

#### Suprapolar cartilage

An additional cartilage in the posterior trabecula-polar region is present in birds. The suprapolar cartilage (Fig. 9), first named by Suschkin [48] as “Supratrabecula”, is situated dorsal to the polar cartilage, and together they form the foramen for the ophthalmic artery (Fig. 9). The suprapolar cartilage appears early in the chondrification sequence, around the time when the polar cartilages and the trabeculae chondrify. The cartilage had an independent origin in the majority of the described birds (*Falco* [48]; *Columba* [67, 68], *Anas* [46, 64], *Struthio* [25, 45, 65], *Phalacrocorax* [61], *Spheniscus* [47], *Euplectes* [50], *Coturnix* [75], *Streptopelia* [54]). In *Gallus* [64, 69], the suprapolar cartilage was fused to the polar cartilage when it appeared, whereas in *Melopsittacus* [49], the suprapolar cartilage was first attached to the ventral surface of the pila antotica (Figs. 7a, d and 10c), before the connection to the polar cartilage was established. In most described birds, the suprapolar cartilage was temporarily connected to the pila antotica/acrochordal cartilage (*Anas* [64], *Struthio* [45], *Spheniscus* [47], *Melopsittacus* [49], *Gallus* [64, 69]).

## The otic region

### Process of development

#### Otic capsule

The otic capsule, which surrounds the anlage of the labyrinth, consists of two parts. The medial cochlear part (Fig. 7a, b) lies lateral to the basal plate and encloses the cochlea, while the lateral canalicular part (Fig. 7a, b) contains the semicircular canals. In the descriptions of *Apteryx* [57] and the common kestrel *Falco* [48], a proliferation of the parachordal cartilage formed a part of the otic capsule. Likewise, Gaupp [59] described for birds the cochlear portion of the otic capsule as part of the basal plate from which the chondrification of the otic capsule originates. He distinguished the formation in birds from the development in other vertebrates, in which the otic capsule also develops in continuity with the basal plate, but the chondrification of the capsule is usually independent of the basal plate. Whereas Goodrich [62] and de Beer [11] stated that the cochlear part of the otic capsule does not form from the basal plate, Sonies [64] emphasized the proximity of the cochlear portion of the otic capsule to the parachordal cartilage, which is much closer in birds than in mammals. He suggested that a coalescence in the mesenchymal or prochondral state may occur, and therefore no discrete anlage of the cochlear portion of the otic capsule is present [64].

In most birds, a continuity between the parachordal cartilage and the otic capsule was described from their first appearance (*Gallus* [58, 64, 69, 74], *Apteryx* [57], *Falco* [48], *Struthio* [25, 65], *Dromaius* [28], *Euplectes* [50], *Melopsittacus* [49]).

Despite this procartilaginous continuity of the two chondrocranial elements, in *Gallus* [64, 69], the cochlear portion could be distinguished from the basal plate, whereas the distinction between the two elements was not possible in the other birds.

In *Phalacrocorax* [61], the mesenchymal anlage of the parachordal cartilage was more advanced than the otic capsule. It was the only stage where a border between the two elements was visible [61]. An independent origin of the cochlear portion was mentioned for *Columba* [67], *Anas* [46, 64], *Spheniscus* [47], and *Streptopelia* [54]. The independent chondrification does not preclude a mesenchymal continuity of the two elements, since incomplete series and different staining can influence the observation of this early embryonic tissue.

#### Chondrification centres

Regardless of the continuous development of the parachordal cartilage and the otic capsule (Fig. 4), in most birds, the otic capsule has two distinct centres of chondrification, one in the cochlear (Fig. 7a), and another in the canalicular part (*Sturnus, Gallus* [64], *Anas* [46, 64], *Spheniscus* [47], *Euplectes* [50], *Coturnix* [75], *Streptopelia* [54]; *Columba* [68]) (Fig. 7a). In *Struthio* [65], no chondrification centre in the cochlear portion was documented. Likewise, some Neognathae had only one chondrification centre. Thus, in *Melopsittacus* [49], the cochlear portion chondrified as a unit with the basal plate, whereas the canalicular portion had an independent origin. In *Gallus*, however, chondrification spread from the cochlear to the canalicular portion [69].

#### Metotic cartilage

Situated ventrally to the canalicular part of the otic capsule, the metotic cartilage (Fig. 7b, c) is a characteristic of birds, whose homology to the subcapsular process of crocodilians (Table 1) is still discussed [16]. In birds, the structure was first mentioned for *Rhea* [56] and *Apteryx* [57] in late embryonic stages and in an early stage of *Falco* [48]. No reference to the development or the cartilaginous state of the metotic cartilage was made before Sonies’s [64] description in *Gallus* and *Anas*. De Beer and Barrington [46] assumed that the metotic cartilage is probably a modification of the cranial ribs. This hypothesis has been rejected based on missing evidence that the two structures are spatially or temporally connected in ontogeny [47, 50, 65, 89].

The development of the metotic cartilage described in literature is inconsistent, and the true nature of its development is indistinct. An independent appearance of the cartilaginous metotic cartilage was only described in Galloanserae (*Gallus* [64] and *Anas* [46, 64]). Both authors used whole-mount staining. In other birds, the metotic cartilage was continuous with the basal plate (*Falco* [48], *Phalacrocorax* [61], *Euplectes* [50], *Melopsittacus* [49]), or continuous with the otic capsule (*Struthio* [25, 65], *Spheniscus* [47, 94]) before being cartilaginous. An experiment by Toerien [94] also indicated an independent origin of metotic cartilage in *Spheniscus*, although it seemed to be originated in connection with the otic capsule.

Intraspecific variation in the sequence of fusion with the basal plate and the otic capsule was observed in *Gallus* [64]. In some birds, the metotic cartilage had two anlagen, one in the basal plate and the other in the canalicular portion (*Euplectes* [50], *Coturnix* [75], the sandgrouse *Pterocles alchata caudacutus* [89], *Passer* [89], *Streptopelia* [89]). Incomplete sampling with missing stages, differences in preparation methods, and unsystematic descriptions complicate the recognition of the development of the metotic cartilage.

#### Fenestra ovalis

The fenestra ovalis is situated in the wall of the otic capsule [47, 50, 69] (Fig. 7b). The formation of the fenestra ovalis was described in the majority of birds as a process of “resorption”. Only Sonies [64] stated that in *Anas* and *Gallus*, the fenestra was formed by incomplete fusion of the cochlear and canalicular part in the region of the stapes (primary origin). Since he used mainly whole mounts, the applied method might be the reason for this difference.

### Chondrification sequence

#### Cochlear portion of otic capsule

In all birds, chondrification of the otic capsule started in the cochlear part. In *Falco* [48], the cochlear portion of the otic capsule was present as early as the acrochordal and the parachordal cartilage, and therefore rather early compared to other birds (Fig. 5), where the same continuous development of the otic capsule with the parachordal cartilage was described. The deviation from the sequence in *Falco* [48] is most likely explained by the chondrification state of the basal plate in the first stage described (“jungen Knorpel”, i.e. “young cartilage”), which is more advanced than in the earliest stages described in the other birds. In *Struthio* [65], the otic capsule also developed in continuity with the parachordal cartilage. Nevertheless, chondrification of the otic capsule started late compared to the other birds (Fig. 6a).

#### Metotic cartilage

The chondrification in the metotic cartilage (Fig. 7b, c) followed in the majority of birds after the onset of chondrification of the otic capsule (Fig. 4). The variation in the onset of chondrification is large (Fig. 5), but does not seem to reflect the different descriptions of metotic cartilage development found in the literature. In *Spheniscus* [47], the first procartilaginous sign in the metotic cartilage appeared at the same stage as the prechordal elements, which is early in the sequence compared to other birds. For the following stage, growth of the cartilage was mentioned, and this stage was taken as proxy for the onset of chondrification, and might be a reason for the relative early appearance of the element in the sequence. In *Struthio* [25, 65], the metotic cartilage appeared late in sequence relative to other birds (Fig. 5), although the anlage of the metotic cartilage was present early. The metotic cartilage remained for quite a long time in a mesenchymal state, and chondrification was only described in later stages [25, 65].

#### Fenestra ovalis

The formation of the fenestra ovalis (Fig. 7b) in *Gallus* [64] took place before the chondrification of the metotic cartilage, whereas the fenestra ovalis was in other birds the last aspect to form in the sequence of the otic region (Fig. 5). In *Gallus*, the differences in the described formation of the fenestra ovalis may have an influence on the sequence. Vorster [69] and Parker [58] described the formation as a process of resorption, whereas Sonies [64] described the fenestra as a fissure that remained after the fusion of the canalicular and cochlear part of the otic capsule. However, in *Falco* [48], although a secondary origin was described, the fenestra ovalis appeared early too, and in *Anas* [64], the described primary origin had no effect on the sequence.

## The orbital region

### Process of development

The orbital region is divided in two parts, the posterior and the anterior orbital cartilages (Fig. 10a), which are temporarily connected by the supraorbital part of the orbital cartilage (Fig. 10b). Only in *Falco* [48], a connection between the intertrabecula and the supraorbital part was described. The reason for this unusual connection is that Suschkin’s [48] terminology named the anterior orbital cartilage together with the supraorbital part “Supraorbitalplatte”, implying a direct connection of the intertrabecula with the supraorbital part, whereas in other birds the anterior orbital cartilage lies between the intertrabecula, or trabecula communis, and the supraorbital cartilage. The detailed description he gave is consistent with the development described in other birds. The middle third of his supraorbital plate becomes resorbed and corresponds to the actual supraorbital part of the orbital cartilage, while the anterior third, that originates from the intertrabecula and is homologous to the anterior orbital cartilage, fuses to the interorbital septum.

#### Posterior orbital cartilage

The posterior orbital cartilage develops in continuity with the pila antotica (Figs. 7a and 10c), which chondrifies as an anterolateral outgrowth of the acrochordal cartilage [11] (Figs. 4 and 7a) and connects the posterior orbital cartilage with the basal plate. The pila antotica was resorbed in ontogeny in *Gallus* [46, 69, 73], but the posterior orbital cartilage remained connected to the basal plate by the pila antotica spuria (Figs. 7a and 10c). In other birds, the pila antotica did not break down completely (*Anas, Passer* [46], *Struthio* [25, 65], *Spheniscus* [47], *Euplectes* [50], *Melopsittacus* [49]) (Figs. 7d and 10c).

#### Anterior orbital cartilage

The anterior orbital cartilage is a laterodorsal extension of the trabecula communis anterior of the interorbital septum [47, 69] (Fig. 10a). It is well developed in early ontogeny, but ‘regresses’ with ongoing development. Its persisting small portion at the dorsal edge of the interorbital septum is the planum supraseptale [21, 46] (Fig. 10c). The planum supraseptale merges posteriorly into the supraorbital cartilage (Fig. 10b). In *Falco* [48], the planum supraseptale was described to form a discrete chondrification. In *Phalacrocorax* [61], variation between individuals in the development of the orbital cartilage were observed that were not related to the regression of the cartilage.

Depending on the description, the anterior orbital cartilage emerged either from the trabecula communis (*Phalacrocorax* [61], *Spheniscus* [47], *Gallus* [69]), the paired trabeculae (*Apteryx* [57], *Anas* [46], *Struthio* [65]), or from a discrete chondrification (the gull *Larus* [46], *Streptopelia* [54]). In other birds, the anterior orbital cartilage seemed to develop in continuity with the interorbital septum (*Melopsittacus* [49, 95], *Euplectes* [50]). In connection with the enlarged eyes [2, 9], the orbital cartilages of birds are reduced compared to non-avian sauropsids.

#### Interorbital septum

The interorbital septum (Fig. 10a, b) is a median structure located anterior to the hypophyseal fenestra between the eyes. Its anterodorsal aspect merges with the anterior orbital cartilage (Fig. 10b). A high interorbital septum (Fig. 10a) is a characteristic of birds. Only *Apteryx* is mentioned as an exception without an interorbital septum [11, 57, 63]. De Beer [11] stated that the formation of the septum is associated with the large size of the eyes. The eyes of *Apteryx* are uncommonly small [57] for a flightless and nocturnal bird [92], but with a night-type retina [96]. The olfactory structures are enlarged [63, 97], so that they are partially located between the small eyes [57]. These adaptions are related to the nocturnal lifestyle which is, in this species, dependent up tactile and olfactory information [92, 96].

Parker [57] described and figured in a chondrocranial stage before the formation of the trabecula communis a structure called “presphenoid” (Table S3, Additional file 3), which in regard to its position (between the eyes) and spatial relation (the “orbito-sphenoid plates” grow out of the presphenoid) [57] (Plate 10, figs. 103,116) corresponds to another feature, to an interorbital septum. The presphenoid was formed of paired plates growing out of the trabeculae and which were initially separated by dense fibrous tissue. In front, the cartilaginous plates were continuous with the unpaired “mesethmoid”; i.e., the nasal septum [91]. Parker [57] may have distinguished between presphenoid and interorbital septum because of the position of the olfactory structure and thus also the nasal septum, which is located between the eyes. The relative position of the interorbital and nasal septum has a direct influence on their origin and, therefore, on their homology. However, Parker [57] himself noted that no clear distinction can be made between “*presphenoid, mesethmoid, septum nasi, and prenasal*” (p. 48).

It was hypothesized that the interorbital septum is formed either from the fused anterior portion of the trabeculae [62], or by fusion of the anterior orbital cartilage [11]. When present, an intertrabecula was described as the base for the formation of the septum (*Falco* [48], *Coturnix* [75]). In birds without an intertrabecula, the septum emerged from the trabecula communis (*Dromaius* [28], *Phalacrocorax* [61], *Spheniscus* [47], *Struthio* [65], *Gallus* [64, 69], *Euplectes* [50], *Melopsittacus* [49, 51, 95]) (Fig. 10a).

Frank [65] and Bellairs [21] described an interorbital septum consisting of three layers. The middle layer was formed by an unpaired mesenchymal condensation and the two lateral ones were formed by the tissue of the anterior orbital cartilage. Chondrification of the interorbital septum seems to start at its lower edge [21, 50].

As in birds, in crocodilians with a reported intertrabecula, the element formed the base of the interorbital septum (*Alligator* [86, 87]), without having previously been in contact with the trabeculae (*Caiman* [19]). In crocodilians without an intertrabecula, the fused anterior part of the trabeculae built the base of the interorbital septum (*Melanosuchus* [72]; *Caiman* [22]).

#### Interorbital fenestrae

**Fig. 11:**
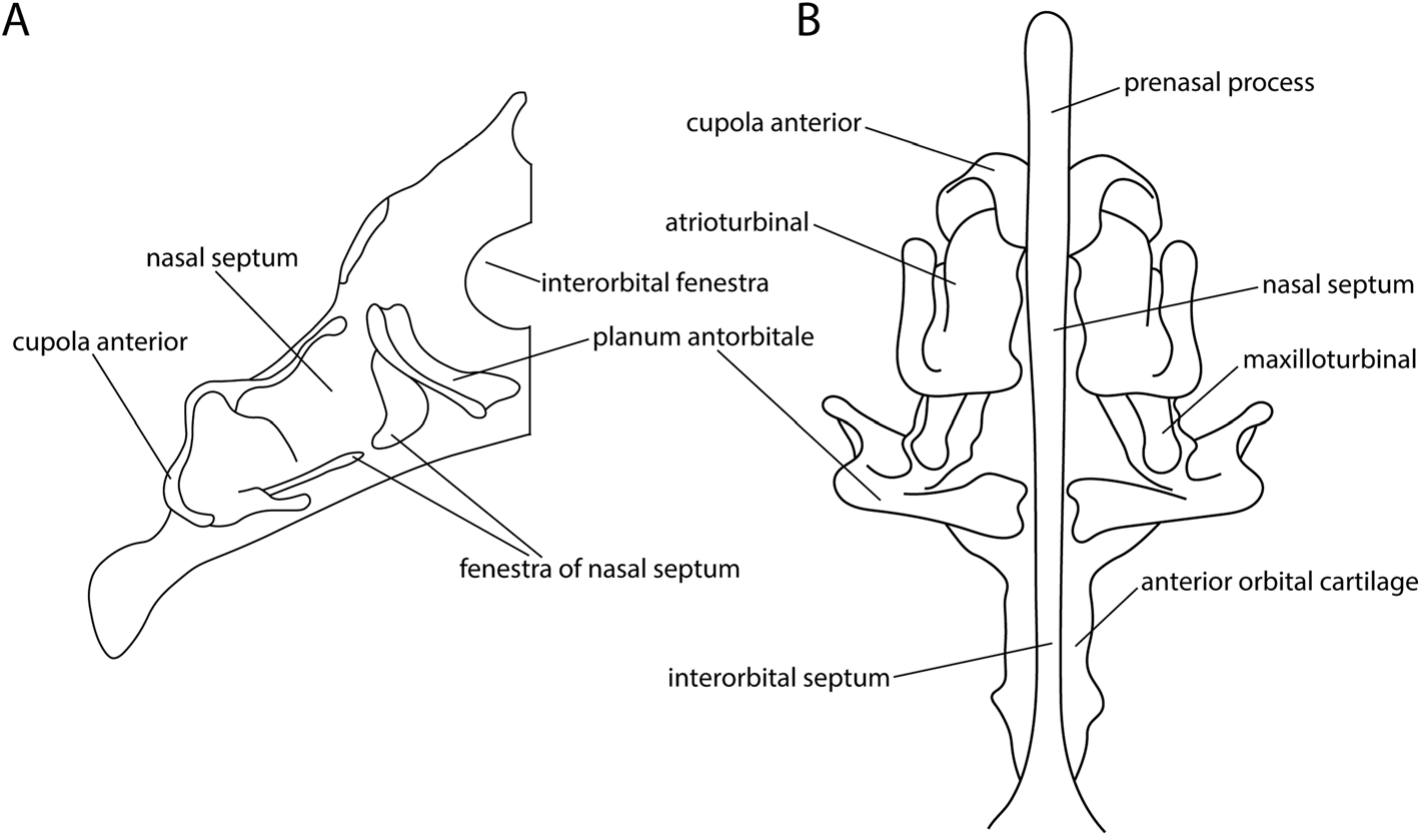
Details of the nasal capsule and turbinals in *Euplectes orix* in **a** lateral view of an older state and in **b** ventral view of an earlier stage of development. Modified from Engelbrecht [50] (stages 7 and 6, respectively)

In most birds, the interorbital septum becomes fenestrated in ontogeny (Figs. 7d and 11). Before the formation of the interorbital fenestra, a thinning of the cartilaginous septum occurs, followed by resorption of cartilage. The fenestra remains filled by a membrane made of the perichondria [69]. In *Melopsittacus*, the missing fenestra may be related to the attachment of a parrot-specific jaw muscle to the interorbital septum [41, 95]. In *Caprimulgus*, Frank [65] described a heavily built interorbital septum without mentioning a fenestra. In *Struthio*, Parker [56] mentioned a thinning in the interorbital septum, whereas Frank [65] stated the fenestra to be absent and Lang [45] did not mention a fenestra.

In crocodilians, the fenestration of the interorbital septum was reported as missing (*Crocodylus* [66], *Melanosuchus* [72], *Caiman* [19]).

### Chondrification sequence

#### Pila antotica

The first cartilage to appear in the orbital region was the pila antotica (Figs. 4 and 7a), as a lateral projection of the acrochordal cartilage (Fig. 7a). In *Coturnix* [75], the pila antotica was recorded when the acrochordal cartilage started to chondrify, without any information about its chondrogenesis. In *Phalacrocorax* [61], the anlage of the pila antotica was already present when the acrochordal cartilage was still mesenchymal. In other birds, the pila antotica followed the onset of chondrification in the acrochordal cartilage (Table 2), since they share the centre of chondrification [44]. In birds, the pila antotica started to chondrify much earlier compared to the ancestral sequence (Fig. 6c).

#### Interorbital septum

The next element that started to chondrify was the interorbital septum (Fig. 10a). It chondrified before the nasal septum (Fig. 7b) in *Gallus* [21, 64], *Spheniscus* [47], and *Phalacrocorax* [61]. In all three, the septa developed from the trabecula communis as in other species (*Struthio* [65], *Euplectes* [50], *Melopsittacus* [49]), where no similar influence on the sequence was evident. In *Falco* [48], the interorbital septum originated from the intertrabecula and started to chondrify before the trabecula communis was formed. Also, in *Coturnix* [75], the interorbital septum developed from an intertrabecula, but without a prior appearance to the trabecula communis. In Neoaves, the interorbital septum chondrified slightly earlier than in Galloanserae.

#### Planum supraseptale and fenestration of the interorbital septum

The chondrification of the planum supraseptale (Figs. 10c) and the fenestra of the interorbital septum (Fig. 7d) followed the interorbital septum. The planum supraseptale appears relative late compared to the ancestral sequence (Fig. 6c). Only in *Falco* [48], the interorbital fenestra was formed before the planum supraseptale appeared (Fig. 5).

## The nasal region

### Process of development

#### Nasal septum

The nasal septum (Fig. 11) is located between the nasal sacs in the nasal capsule (Fig. 7d). It was described to emerge either from the dorsal edge of the trabecula communis (*Gallus* [64, 69], *Euplectes* [50], *Melopsittacus* [49, 51]), or as an outgrowth of the intertrabecula (*Falco* [48], *Coturnix* [75]). In Palaeognathae, the fenestrae in the nasal septum (Fig. 11a) were absent (*Struthio* [45, 65] or not reported (*Apteryx* [57], *Dromaius* [28, 45, 56], *Rhea* [45, 56]).

In crocodiles, the nasal septum developed as an outgrowth of the trabecula communis (*Caiman* [19, 22]), and was not fenestrated (*Melanosuchus* [72]).

#### Prenasal process

The prenasal process (Fig. 7a) lies anterior to the nasal capsule and cupola anterior (Fig. 11b) and is thus the most anterior part of the chondrocranium. Posteriorly, it is continuously connected to the nasal septum. The feature is shared by birds and crocodilians (Table 1). The presence of a prenasal process in birds is explained by a posterior displacement of the cupula anterior and the elongated premaxilla [50]. The only exception is *Apteryx* [50], whose external nares are positioned at the tip of the beak [50, 57, 63, 92, 98], and therefore, a prenasal process is mentioned to be missing [50]. In contrast, de Beer [11] found that the nasal septum in *Apteryx* takes the position of the prenasal process in other birds. Parker [57] himself described a “prenasal cartilage” in *Apteryx*, although he also described the nasal capsule extending from the turbinal region to the tip of the beak. The prenasal process is either described as a prolongation of the trabecula communis (*Phalacrocorax* [61], *Spheniscus* [47], *Euplectes* [50], *Melopsittacus* [49], *Gallus* [64, 69]), or as a prolongation of the nasal septum (*Apteryx* [57], *Anas* [46], *Struthio* [25], *Coturnix* [75]). In crocodilians, which have the external nares like *Apteryx* at the tip of their snout, the prenasal process is small [11, 22, 86, 93].

#### Turbinals

Three turbinals, the atrio- and maxilloturbinal (Fig. 11b) and the concha nasalis (Fig. 7d), are recognized in birds [46]. The atrioturbinal is located in the anterior region of the nasal capsule. At the posterior end, it merges continuously into the maxilloturbinal. The concha nasalis is the most posterior of the three turbinales. In *Opisthocomus*, Bang [99] described that the atrioturbinal (“anterior concha”) was missing, whereas Parker [83] mentioned that the atrioturbinal (“inferior turbinal”) was simpler but otherwise similar as in *Gallus*. The concha nasalis is missing in several smaller passerine species (the warbler *Sylvia*, the wren *Troglodytes*, the finch *Fringilla* [100], *Passer* [51], the swallow *Hirundo* and swifts [99]). Schultze [100] implied a relation of the presence of the concha nasalis to the size of the bird since the crow *Corvus* and the thrush *Turdus* [100] have a small concha, and Lang [51] described the arrangement of the turbinals in relation to the space depending on the dimension of the eyes and the nasal capsule. In *Struthio*, the turbinals were described to be arranged in line, whereas in *Passer* and *Melopsittacus*, they were arranged one above the other [51]. For birds with relatively large eyes as *Struthio*, *Rhea*, and *Caprimulgus*, a dorsal position of the concha nasalis to the planum antorbitale was described [65]. De Kock [49] assumed a correlation with the presence of the paranasal cartilage that gave rise to the concha nasalis in some birds (*Gallus* [64], *Anas* [46], *Struthio* [65]). This was questioned by Vorster [69], who reported a concha nasalis and the lack of the paranasal cartilage in *Gallus*.

#### Planum antorbitale

**Fig. 12:**
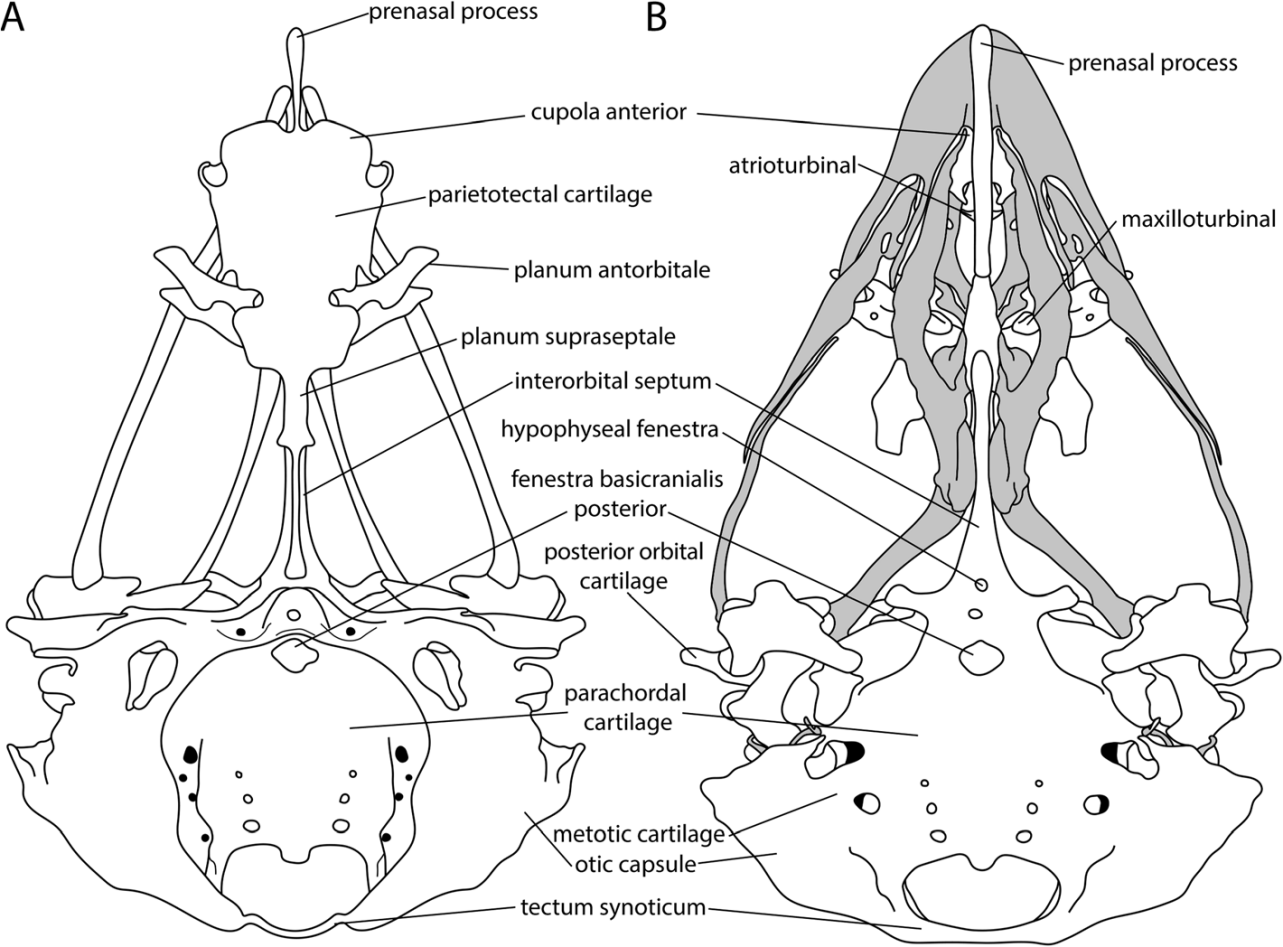
Late chondrocranial stage of *Euplectes orix*. **a** dorsal, and **b** ventral view (including dermal bones of the palate in grey). Modified from Engelbrecht [50] (stage 8)

The planum antorbitale (Fig. 7b, c) develops between the eyes and the nasal capsule (Figs. 7b and 11), forming a boundary between the orbital and nasal regions that closes the nasal capsule posteriorly. Exceptions are *Struthio* [45, 65] and *Apteryx* [57] where the nasal capsule, and therefore the planum antorbitale, lies partly between the orbits. Various patterns of development are described in the literature [49]. Either the planum antorbitale develops from the parietotectal cartilage (*Spheniscus* [47], *Gallus* [69]) (Figs. 7b and 12), had its origin in the anterior orbital cartilage (*Melopsittacus* [49]), formed from the trabeculae (*Falco* [48], *Gallus* [64]), or developed from the paranasal cartilage (*Streptopelia* [44]). Even the development from an independent chondrification centre was described (*Anas* [46], *Euplectes* [50], *Coturnix* [75]). In *Spheniscus* [47], only a rudimentary planum antorbitale was developed and formed an incomplete posterior wall. In *Rhea* and *Dromaius* [45], an independent planum antorbitale was missing. Lang [45] assumed its fusion with the side wall of the nasal capsule due to the enlarged eyes. In *Struthio*, the planum antorbitale was well-developed, but narrow and, also, in close contact with the side wall of the capsule [45]. In contrast to crocodilians [11], a cupola anterior (Figs. 11 and 12) is present in birds.

### Chondrification sequence

#### Nasal septum

The nasal septum (Fig. 7a) was the first cartilage in the nasal region that started to chondrify (Fig. 5, Table 2), following the prechordal elements. In *Falco* [48], the nasal septum chondrified before the trabecula communis due to its origin from the intertrabecula, which was present earlier than the trabecula communis. In *Coturnix* [34, 75], the nasal septum appeared not earlier than in other species although an intertrabecula was described. In Galloanserae, the nasal septum and the prenasal process chondrified later than in Neoaves (Fig. 6b). The late appearance of the nasal septum in *Meleagris* [37] can be explained by the described stage of development, which is already well advanced.

#### Prenasal process

The next element that chondrified was the prenasal process (Fig. 7a). The variation in the appearance of the prenasal process is large (Fig. 5), although there is not much variation in the development. In *Phalacrocorax* [61] and *Spheniscus* [47], the process was described as a prolongation of the trabecula communis. In both birds, the prenasal process appeared earlier than in birds with the same described development. In *Coturnix* [75], the process was described as an extension of the nasal septum, and appeared late compared to the stages described and photographed by Nakane and Tsudzuki [34], and compared to birds with the same described development. The difference in the described mode of formation of the prenasal process (Table 3) seems not to influence its relative early or late appearance (Fig. 5). In *Meleagris*, the relative late appearance is an issue of the selected stages [37] and missing information [33], as these studies focused on ossification and not chondrification.

**Table 3:** Character matrix on the differences in the described development mode of elements (Fig. 13a). **Character 1**: origin of the fenestra basicranialis posterior: 0 = absent; 1 = primary origin; 2 = secondary origin;? = no information. **Character 2**: formation of trabecula communis: 0 = by fusion with intertrabecula; 1 = by fusion of trabeculae;? = no information. **Character 3**: chondrification centres of otic capsule: 0 = centre in cochlear portion; 1 = centre in cochlear and canalicular portion; 2 = centre in canalicular portion;? = no information. **Character 4**: cartilaginous metotic cartilage: 0 = not independent, but unclear to which part of the chondrocranium they are fused; 1 = independent; 2 = fused to basal plate; 3 = fused to otic capsule;? = no information. **Character 5**: development of anterior orbital cartilage: 0 = independent origin; 1 = from trabeculae; 2 = from trabecula communis; 3 = in continuity with interorbital septum;? = no information. **Character 6**: base of interorbital septum: 0 = intertrabecula; 1 = trabecula communis;? = no information. **Character 7**: base of prenasal process: 0 = nasal septum; 1 = trabecula communis;? = no information. **Character 8**: base of nasal septum: 0 = intertrabecula; 1 = trabecula communis;? = no information

Characters	1	2	3	4	5	6	7	8
Species								
*Apteryx australis*	1	?	?	0	1	?	0	?
*Dromaius novaehollandiae*	0	?	?	?	?	1	?	?
*Rhea americana*	?	?	?	0	?	?	?	?
*Struthio* sp.	?	1	2	3	1	1	0	?
*Coturnix japonica*	?	0	1	?	?	0	0	0
*Meleagris gallopavo*	?	?	?	?	?	?	?	?
*Gallus gallus*	1/2	1	0	1	2	1	1	1
*Anas platyrhynchos*	1	1	1	1	1	?	0	?
*Caprimuglus pectoralis*	?	?	?	?	?	?	?	?
*Columba livia*	2	?	1	?	?	?	?	?
*Streptopelia senegalensis*	2	?	1	?	0	?	?	?
*Opisthocomus hoazin*	0	?	?	?	?	?	?	?
*Spheniscus demersus*	0	1	1	3	2	1	1	?
*Larus* sp.	?	?	?	?	0	?	?	?
*Phalacrocorax carbo*	1	1	?	2	2	1	1	?
*Falco tinnunculus*	2	0	?	2	?	0	?	0
*Melopsittacus undulatus*	0	1	2	2	3	1	1	1
*Euplectes orix*	2	1	1	2	3	1	1	1
*Passer* sp.	?	?	?	?	?	?	?	?
*Sturnus* sp.	?	?	1	?	?	?	?	?
*Hirundo rustica*	?	?	?	?	?	?	?	?

#### Parietotectal cartilage and planum antorbitale

The parietotectal cartilage and the planum antorbitale (Fig. 12) were next in sequence followed by the concha nasalis. The variation in the onset of chondrification of the planum antorbitale is relatively large, the reason might be the disparity in the described developmental pattern of this element in literature [49]. In *Falco* [48], intercellular substance in the planum antorbitale appeared early compared to other birds and in the next described stage the planum antorbitale was well developed.

#### Fenestration of the nasal septum

The fenestrae of the nasal septum formed in most birds before the interorbital fenestra (Fig. 11a). Only in *Falco* [48] (Fig. 5), the fenestra of the interorbital septum preceded the fenestration of the nasal septum.

#### Maxilloturbinal

The next elements of the nasal region that started to chondrify were the maxilloturbinal (Figs. 7d and 11b) and the nasal capsule (Fig. 7d).

#### Atrioturbinal and cupola anterior

The atrioturbinal and the cupola anterior (Fig. 11b) were the last elements of the avian chondrocranium that chondrify (Fig. 5, Table 2). In contrast to the sequence of turbinals in other birds, in *Coturnix* [75], the atrioturbinal appeared before the other turbinals. The order of sequence is in most birds: first the concha nasalis, followed by the maxilloturbinal, and last the atrioturbinal. In *Melopsittacus* [49], the atrioturbinal was the last element in the sequence, whereas in the other birds, it was the cupola anterior (Fig. 5, Table 2).

## The occipital region

### Process of development

In birds, the roof of the chondrocranium is reduced to a strip of cartilaginous structures that form the posterodorsal border of the foramen magnum. The so-called tectum synoticum (Figs. 7c and 12), which connects the otic capsules, is occasionally associated with a tectum posterius (Fig. 4) that joins the occipital arches [11] (Fig. 7b, c). The distinction between the two tecta is not absolute [11], because of rather unspecific boundaries [101]. In the literature, considerable variation in the described formation of the chondrocranial roof in birds can be found [11, 101].

#### Tectum synoticum

The tectum synoticum is formed without any connection to the otic capsules or occipital arches from paired chondrifications (*Anas* [64, 101]*, Gallus* [64, 101], *Spheniscus* [101]) or as one unpaired element (*Falco* [48], *Phalacrocorax* [61]). In many birds, however, the tectum was described to be continuous with the otic capsules from its first appearance (*Gallus* [69, 73], *Struthio* [25, 65], *Spheniscus* [47], *Euplectes* [50]). In *Anas* [46, 101], the tectum synoticum appeared later in development, is discontinuous in its median portion, but remains connected to the otic capsules on both sides. In *Gallus*, a deep notch in the dorsal midline of the tectum synoticum was described in a relative late stage [69], but without becoming discontinuous.

#### Tectum posterius

A tectum posterius (Fig. 4) was mentioned in several bird species except in Galloanserae. Lang [51] mentioned for *Passer*, additionally to the tectum synoticum, an independent tectum posterius without detailed information on its chondrogenesis. In *Phalacrocorax* [61], the tectum was connected to the canalicular part by condensed mesenchyme, and later also to the occipital arch. Slabý [61] could not tell with certainty whether it was a tectum synoticum or posterius. In *Struthio* [65], combined precartilaginous tecta with an uncertain extent were described. The tectum posterius was described to be well developed, while the tectum synoticum was incomplete, and still the possibility that the tectum posterius was a part of the tectum synoticum was not precluded. De Kock [49] described for *Melopsittacus* the difficulty to ascertain the extant of the mesenchymal, diffuse mass that formed the tectum posterius. However, he mentioned that the mass was mainly connected with occipital arches, although the mesenchymal mass was in contact with the otic capsule as well.

Lang [51] mentioned for *Melopsittacus* only a tectum synoticum continuous with the otic capsule. In *Streptopelia* [55], the tectum was mainly formed by the coalescence of the occipital arches, although the contribution of the otic capsule to its formation was mentioned. Vorster [69] did not explicitly mention a tectum posterius but observed in *Gallus* that the ventral root of the tectum synoticum spreads over the occipital arch. He clarified that this attachment cannot be seen as distinct element. Experiments on the development of the chondrocranial roof in birds, involving removal of the ear placodes and occipital processes [101], indicated that the roof develops independently from the otic capsule and occipital arches, and that the tectum posterius is not a discrete cartilage but instead a part of the tectum synoticum.

Most authors mentioned difficulties in defining the exact extent of the tectum and its mesenchymal connections. The occurrence of intraspecific differences among the descriptions of the different studies may indicate that methodological aspects such as stage selection and staining have influenced the results.

#### Further cartilaginous roof elements

Among birds additional cartilaginous roof elements dorsal to the tectum synoticum have been described in *Falco* [48] (“Epiphysenknorpel”) and in *Passer* [51] (“Rudimente einer Parietalplatte”). In crocodilians, the occipital arch seems to contribute to the tectum (*Melanosuchus* [72], *Crocodylus* [23]).

#### Cranial ribs

In some birds, in the occipital region of the parachordal cartilage, vestigial cranial ribs were described on the ventral aspect of the basal plate (Fig. 7a). They appear early in ontogeny around the onset of chondrification of the basal plate and disappear shortly after. Cranial ribs were first mentioned in chicken embryos by Froriep [102] and documented thereafter in several other bird species. In some birds, the described cranial ribs were mesenchymal (*Falco* [48], *Phalacrocorax* [61], *Spheniscus* [47], *Melopsittacus* [49], *Gallus* [69, 74]), in others intercellular substance was observed (*Anas* [46], *Struthio* [65], *Gallus* [51], *Melopsittacus* [51], *Euplectes* [50], *Streptopelia* [89], *Pterocles* [89], *Passer* [89]).

No cartilaginous cranial ribs were observed in the studied stages of *Dromaius* [28] and in *Gallus* and *Anas* studied by Sonies [64], who defined ribs as cartilaginous skeletal elements only. The true nature of the cranial ribs is still unclear [65, 89]. They were mostly described as being attached to the ventral surface of the occipital basal plate [48, 51, 65, 69], situated between the myomers [47, 49, 50, 69], and being continuous with prolongations of the mesenchymal myocommata [49, 50]. An association with occipital hypocentra was only mentioned by de Beer and Barrington [46] and by Zaher and Riad [89]. Cranial ribs are reported for at least one non-avian sauropsid, i.e. the red-bellied short-necked turtle, *Emydura subglobosa*, too [103].

### Chondrification sequence

#### Tectum synoticum

The tectum synoticum appeared as one of the last elements of the chondrocranium (*Struthio* [65], *Spheniscus* [47], *Coturnix* [34]) (Fig. 5), when chondrification in the nasal region had already started and dermal ossifications were present (*Melopsittacus* [49], *Gallus* [69]). In *Gallus* [69], *Euplectes* [50] and *Phalacrocorax* [61] the tectum synoticum seems to start earlier than in other birds. In *Euplectes* [50], the completely chondrified state of the tectum was not mentioned, only the onset of chondrification. In *Spheniscus* [47], *Melopsittacus* [49], and *Gallus* [69], several cartilaginous states were described, and in all of them, the tectum was cartilaginous at the same time as chondrification of the atrioturbinal started. In case the two elements chondrify also in *Euplectes* [50] around the same time, the chondrification sequence of the tectum would be the same as in the other birds.

## Discussion

### Methodological and evolutionary considerations

#### Methodological considerations

**Fig. 13:**
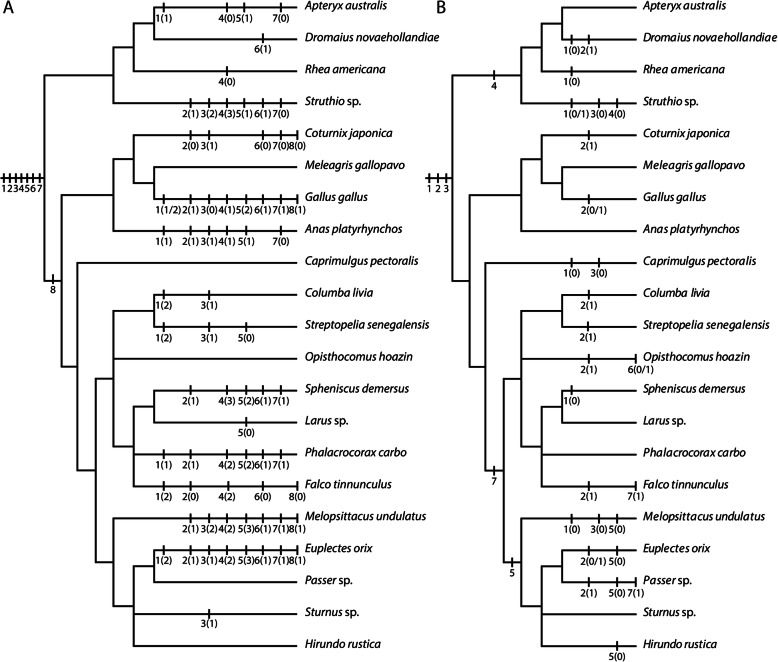
Distribution of chondrocranial diversity in 21 bird species as atomized here in 15 characters. The seeming inconsistence in the mapping of the characters in the tree is based on the fact that many parts of the tree are unresolved or that the ancestral condition is uncertain. **a** Pathways of development (Table 3) and **b** Presence/absence of chondrocranial elements (Table 4). Elements on the tree result from character mapping as described in the development process

We present a comprehensive analysis of morphological variation of chondrocranial structures (Fig. 13, Tables 3 and 4). When making our literature survey, we faced several difficulties in using data from different centuries and authors, and we carefully evaluated each character before coding. The methods and the dyes (Table S1, Additional file 1), the selected stages (Table S4, Additional file 4; Table S5, Additional file 5), the differences in the definition of chondrocranial elements and the terminology used in the past (Table S2, Additional file 2; Table S3, Additional file 3): all these factors can cause problems when comparing the chondrocranial development of different species. The method used influences how different modes of development and anatomical specializations or commonalities are identified. The effect of the methods was discussed almost from the earliest descriptions on [48, 64]. The consequence of diverging methodological approaches is also known from other groups and was discussed for squamates by Yaryhin and Werneburg [12].

**Table 4:** Character matrix for the presence or absence of elements (Fig. 13b). **Character 1**: fenestra basicranialis posterior: 0 = absent; 1 = present; 0/1 = contradicting information;? = no information. **Character 2**: intertrabecula: 0 = absent; 1 = present; 0/1 = contradicting information;? = no information. **Character 3**: fenestra of interorbital septum: 0 = absent; 1 = present;? = no information. **Character 4**: fenestra of nasal septum: 0 = absent; 1 = present;? = no information. **Character 5**: concha nasalis: 0 = absent; 1 = present;? = no information. **Character 6**: atrioturbinal: 0 = absent; 1 = present; 0/1 = contradicting information;? = no information. **Character 7**: additional tectal cartilage: 0 = absent; 1 = present;? = no information

Characters	1	2	3	4	5	6	7
Species							
*Apteryx australis*	1	0	?	?	1	1	0
*Dromaius novaehollandiae*	0	1	1	?	?	1	?
*Rhea americana*	0	?	1	?	1	1	?
*Struthio* sp.	0/1	0	0	0	1	1	0
*Coturnix japonica*	?	1	?	1	1	1	?
*Meleagris gallopavo*	?	?	1	?	?	?	?
*Gallus gallus*	1	0/1	1	1	1	1	0
*Anas platyrhynchos*	1	0	1	1	1	1	0
*Caprimulgus pectoralis*	0	?	0	1	1	1	?
*Columba livia*	1	1	?	?	?	?	0
*Streptopelia senegalensis*	1	1	1	1	1	1	0
*Opisthocomus hoazin*	?	1	1	?	1	0/1	?
*Spheniscus demersus*	0	0	1	1	1	1	0
*Larus* sp.	1	?	?	?	?	?	?
*Phalacrocorax carbo*	1	0	1	?	?	1	0
*Falco tinnunculus*	1	1	1	1	?	1	1
*Melopsittacus undulatus*	0	0	0	1	0	1	0
*Euplectes orix*	1	0/1	1	1	0	1	0
*Passer* sp.	1	1	1	?	0	1	1
*Sturnus* sp.	1	0	?	?	?	?	?
*Hirundo rustica*	?	?	?	?	0	?	?

In the developing skull, from the earliest mesenchymal anlagen to the first ossifications, different cartilaginous states occur in parallel and distinct borders between the developing elements and the surrounding tissue do not exist [24, 42, 48]. Within a chondrocranial element, different states of chondrogenesis can be present [12, 15]. In the descriptions of the early stages, mesenchyme is often depicted together with cartilage in a uniform way, and only the occurrence of an element is mentioned instead of a precise description of its formation. Moreover, the process of transformation from mesenchyme to hyaline cartilage is often not described and only the growth of the element is stated. However, it is important to know the detailed development of the chondrocranium from the earliest anlagen to the presence of hyaline cartilage in order to understand its developmental process properly [15, 24, 42]. We avoid this difficulty by considering only elements in which chondrification has been explicitly mentioned. This may mean that the appearance of certain elements in some species may be in reality earlier than reported in our survey.

Terminology and definition have an influence on how the descriptions are understood and interpreted, leading to ambiguities. In one of the earliest descriptions of the development of a bird chondrocranium, the need for changes of the terminology used in earlier work was already pointed out [58]. We tried to minimize this problem by carefully checking the terms and their usage and being transparent by providing a compilation of the different terms (Table S2, Additional file 2; Table S3, Additional file 3). If the use of terms or the interpretation of a structure was unclear, we refer to the original terminology and the original publication. In the case of the processes of the trabecular region we did not consider them in the chondrification sequence, because of the unclear homology.

Many different staining procedures [12] have been used in the studies on skull development in birds (Table S1, Additional file 1) and make it difficult to compare the development among different bird species and significantly change the actual impression of existing structures and their development. An additional uncertainty factor is that it is not known for which stages of a series which staining or, in the case of *Anas* [46] which method was used. Deviating results between whole-mount and histological methods were already mentioned by Sonies [64]. This issue could not be avoided, because of the use of data from different sources. In cases with contradictory results, where we assumed that staining might have an influence or where this was already discussed, we mentioned this in the text. Data from sources without a detailed description of the staining were treated more carefully.

Incomplete ontogenetic series, or even the selection of sections and the section thickness [12], can lead to false conclusions about development, since the formation of an element or fenestra is hardly traceable when transitional stages are missing [48]. We were not able to circumvent the lack of comparable stages, since we used the data from different sources. But circumvented the issue by using only data for species for which at least four stages have been described and only considered chondrocranial elements when they were reported at least in six species.

Differences in the descriptions and methods used pose great challenges to extracting with certainty some morphological details from the old (often German) literature. Clearly new revisionary work is needed to asses some characters and ontogenetic pathways of change.

#### Morphology and process of development

To analyse the variation among bird chondrocrania in a phylogenetic framework, we mapped the differences of the chondrocranium and the diverging modes of structure development (Tables 3 and 4) on a given molecular tree (Fig. 13).

Importantly, much of the variation lies in the formation of certain structures (Fig. 13a, Table 3). Only a smaller part of the variation in the static chondrocranial morphology of birds relates to the absence of fenestrae and turbinals, and few variation relate to the presence of additional elements such as the intertrabecula or roof elements (Fig. 13b, Table 4).

Differences in chondrocranial morphology across birds also concern the shape and proportion of structures. The compilation of chondrocrania (Fig. 1), most of all the last figured stages on the right side, indicates the presence of shape variation being present in embryonic skulls. This is as exemplified by the case of *Apteryx*. The chondrocranium of *Apteryx* shows several proportional changes in the orbital and nasal region when compared to its closest relatives and are related to unique features in the bony skull – best interpreted by the adaptations of the associated sensory organs to its lifestyle. The exceptional morphology of the orbitonasal region of *Apteryx* makes it possible to identify the differences in shape and proportions, which are otherwise not easy to capture with descriptions and are therefore not given much consideration. The derived morphology of the adult (Fig. 3) is already reflected in specializations of the embryonic skull.

We detected apomorphies to discriminate between Palaeognathae and Neognathae. These include the missing fenestrae in the nasal septum of the palaeognaths (Fig. 13b, Table 4) and the secondary origin of the basicranial fenestra restricted to neognaths (Fig. 13a, Table 3). Characters to differentiate between Galloanserae and Neoaves include the presence of a distinct cartilaginous metotic cartilage in Galloanserae, while in most Neoaves the metotic cartilage was continuous with the basal plate at its ontogenetic appearance (Fig. 13a, Table 3). Some of the patterns may simply reflect missing information about species. Similar caution and testing are required for some patterns of less inclusive clades such as in the formation of anterior orbital cartilage from the trabeculae and the prenatal process from the nasal septum, which have only been described in palaeognaths and some Galloanserae, as well as the case of the missing concha nasalis in Passeriformes (Fig. 13b, Table 4), which is limited to smaller species.

#### Chondrification sequence

The pattern of chondrification is generally similar among birds (Fig. 5, Table 2). However, variation was found in all regions, though the sequence in the peri- and prechordal region in the base of the cranium is more conserved, and the last elements that chondrify are in all species the most anterior ones [24].

Differences in the basal plate, including the mode how the basicranial fenestra is formed, characterizes several species, whereas the variation in the chondrification pattern is only aberrant in *Phalacrocorax* [61] (Fig. 5). In the otic region, variation in the onset of chondrification among birds was found in all elements. The large range in the onset of chondrification reconstructed for the metotic cartilage is influenced by the condition coded for *Struthio* [25, 65] and *Spheniscus* [47], in which, respectively, the element chondrifies relatively late or relatively early (Fig. 5).

Variation is present in the orbital and nasal elements. In contrast to other birds, in *Falco* [48], the interorbital and the nasal septum appeared before the trabecula communis and the fenestra of the interorbital septum formed much earlier and before the fenestration of the nasal septum (Fig. 5). In the orbital and nasal septum and the prenasal process, there is only little variation in the described formation. In the case of the prenasal process, *Coturnix* [75] and *Meleagris* [33, 37] contribute much to the range of timing indicated in the reconstructed sequence (Fig. 5). The relative late appearance in these birds is most likely explained by the selection of the stages and missing information on chondrogenesis. The early appearance of the atrioturbinal before the other turbinals in *Coturnix* [75] (Fig. 5) is unique among birds. In the planum antorbitale, the incongruence of the described formation can have an influence on the sequence, but the early onset of chondrification of this element in *Falco* [48] (Fig. 5) also contributes to the range of timing. In *Struthio* [25, 45, 56, 65], as a representative of the Palaeognathae, chondrification in the elements of the otic region occurred later than in Neognathae (Fig. 6a), while the majority of the other elements chondrified earlier. Perhaps the late chondrification of the otic region is a pattern that only holds for *Struthio*.

Palaeognathae are characterized by the missing fenestration of the nasal septum (Fig. 6a) and, if present, a basicranial fenestra of primary origin. The prenasal process develops in continuity with the nasal septum, and the anterior orbital cartilage develops in continuity with the trabeculae. In Neognathae, fenestrae in the nasal septum are present and a secondary origin of the basicranial fenestra is described only in this group.

The sequence was similar in Galloanserae and Neoaves, except that in Neoaves the majority of the elements chondrified slightly earlier (Fig. 6b). Galloanserae are characterized by fenestrations in the interorbital and nasal septum, as well as the presence of a basicranial fenestra. Only in this group there is a stage in which an independent cartilaginous metotic cartilage occurs (Fig. 13a, Table 3). In Neoaves, the fenestrae of the nasal septum are present, whereas those of the interorbital septum are missing in some species (Fig. 13b, Table 4). Only in this group turbinals are described to be missing, such as the concha nasalis in Passeriformes (Fig. 13b, Table 4) and the atrioturbinal in the hoazin (Fig. 13b, Table 4). And only Neoaves additional tectal elements are described (Fig. 13b, Table 4).

Compared to non-avian sauropsids, is the relative early onset of chondrification in the basal plate, the early chondrification of the pila antotica, and the late chondrification of the planum supraseptale a pattern for birds (Fig. 6c). The onset of chondrification in the prechordal region has been documented in some crocodiles and squamates, but never in birds.

## Conclusion

In this study we reviewed the development of the chondrocranium in 21 birds and compared the chondrification sequence of ten birds and five non-avian sauropsids. Although the morphology of the chondrocranium is thought to be conservative in vertebrates in general and among sauropsids or birds, variation in the development, presence, and shape of elements does exist. Differences between non-avian sauropsids and birds can be explained by the development of the chondrocranium in close connection with the sense organs and the brain [12, 17]. In birds, structures as the high interorbital septum or the reduced orbital cartilages correlate with large eyes, and the reduced tectum synoticum with the relatively large brain. Chondrocranial variation among birds is present but relates to developmental patterns rather than the presence or absence of specific elements. The use of molecular markers and neural crest cells [82] can help provide answers to open questions, such as the presence of an intertrabecular. But this does not circumvent the need for comparative anatomical considerations such as defining at what point a procartilaginous structure is considered an independent chondrocranial element. In the case of divergent developmental patterns described in the literature for homologous chondrocranial structures, it often remains uncertain whether these are true differences or just methodological artifacts. So clearly more empirical studies are needed to revise previous reports.

Notwithstanding the existing chondrocranial variation, it remains difficult to assign patterns to specific avian clades. Only three chondrocranial features were found that might allow a distinction among groups. Regarding the diversity in shape and proportion of the embryonic skull, the striking example of the kiwi shows that these are already present in the chondrocranium. To quantify shape differences, methods such as geometric morphometrics, rarely applied to chondrocrania [104, 105], would be appropriate.

During early development of the chondrocranium, the chondrification sequence is similar among birds, with the exception of the cormorant, but differs from that of other reptiles. In the progression of chondrification, there are differences between the birds in the otic, orbital, nasal, and occipital regions. However, a correlation to morphological variation could not be found.

Comparative analytical evaluation of comparative developmental data is important to understand how morphological diversity evolves through the interaction of ontogeny and phylogeny. The reviewed data from the long-established field of descriptive research on chondrocranial anatomy certainly benefits from the incorporation of a phylogenetic framework and of quantitative analyses of ontogenetic trajectories and of shape.

## Material and methods

We codified the onset of chondrification in ten bird species and in five species of non-avian sauropsids that serve for outgroup comparison (Fig. 2). Only characters for which there is no ambiguity in homologization were considered. Data were used when at least four stages were described, and chondrocranial characters were analyzed when they were documented for at least six bird species [29]. Missing data are indicated with a “-” when a character does not develop in the species, or with a “?” when a character is not mentioned in the description. In total, 23 characters were analyzed. The chondrocranial features were coded as present when authors reported first signs of chondrification or mentioned the character as being cartilaginous, or when a cartilaginous feature was illustrated, whereas fenestrations were coded as present when they were reported to be formed, or when formation through resorption started. Characters were excluded when only information on mesenchyme was available. The simultaneous appearance of chondrocranial characters most likely reflects in many cases the low resolution of series. Variation in the sequence can potentially be biased because of reconstruction of the sequence from different sources (consensus “species”), or by differences in the preparation method. Issues of intraspecific variation may not be fully considered in the dataset, but our study does provide a critical and conservative overview for birds.

To study and reconstruct the chondrification sequences, we applied the continuous analysis method [106] in Mesquite version 3.61 software. It uses developmental timing data (Table 2) scaled between “0” (conception) and “1” (last element to appear) and, using parsimony, it optimizes these continuous scores on a given tree (Fig. 2) (see also [31]). The Neornithes consensus line represents the mean values of the individual chondrification events of all ten bird species ordered in ascending order of relative timing of chondrification.

The phylogenetic framework (Fig. 2) is a consensus of several molecular studies based on http://www.timetree.org/ [access: 2020-05-13]. It presents a consensus of several molecular studies. Birds are grouped in Palaeognathae and Neognathae. Neognathae includes the monophyletic clade Galloanserae consisting of Anseriformes and Galliformes [107], sister group to Neoaves [108, 109]. The majority of bird species belong to the latter.

For the analysis of chondrocranial diversity the data of 21 species were recorded for seven discrete chondrocranial elements (Table 4) and for developmental differences in eight elements (Table 3). The phylogenetic distribution was studied using a parsimony-based state reconstruction running on the Mesquite version 3.61 software and visualized on a phylogenetic framework from http://www.timetree.org/ [access: 2020-05-12]. In case of unresolved nodes of the tree, the ancestral state is reconstructed as uncertain and potential apomorphies of the ground pattern are shifted to more terminal nodes in the tree.

The used nomenclature in the study follows Sonies [64], de Beer [11], and Vorster [69]. For homologous structures, various anatomical terms were used by authors in the last two centuries (Table S3, Additional file 3).

## Supplementary Information

**Additional file 1: Table S1.** Chondrocranial development sources on bird and reptile outgroups used in this study.

**Additional file 2: Table S2.** Terminology of the processes of the trabecular-polar region and their presumed homology [25, 47, 48, 50, 64].

**Additional file 3: Table S3.** Selection of anatomical terms used for homologous chondrocranial characters in the bird chondrocranial literature.

**Additional file 4: Table S4.** Compilation of the original stages of species used for the analysis of the chondrification sequence. Continued in Table S5, Additional file 5.

**Additional file 5: Table S5.** Compilation of the original stages of species used for analysis of the chondrification sequence. Continued from Table S4, Additional file 4.

## Data Availability

All data generated or analyzed during this study are included in this published article.
