# Supplementary material for: Evolution and development of the bird chondrocranium
Source: Front Zool. 2021 Apr 29;18:21. doi: 10.1186/s12983-021-00406-z (PMC8082637; doi:10.1186/s12983-021-00406-z)
Supplement: Supplementary file 1 — Additional file 1: Table S1. Chondrocranial development sources on bird and reptile outgroups used in this study. [file 12983_2021_406_MOESM1_ESM.docx]

**Table S1** Chondrocranial development sources on bird and reptile outgroups used in this study.

| Species | Reference | No. of stages | Range of stages | Material | Staining method | Section thickness |
| --- | --- | --- | --- | --- | --- | --- |
| *Struthio camelus* | Lang [45] | 3 | St. A-C (44 - 70mm)  (head length) | serial sections | Azan by Heidenhain | 30 µm & 40µm |
| *Struthio camelus* | Parker [56] | 3 | St. A-C (1.5 - 2.5 inches) (head) | serial sections | * | * |
| *Struthio* sp. | Brock [25] | 4 | 10.5 - 21mm | serial sections | *in toto:* borax-carmine, or alum cochineal & Orang G | 10, 25, 50 µm |
| *Struthio* sp.  "ostrich" | Frank [65] | * | 3.3 - 34d | serial sections | *in toto:* Mayer's acid haemalum & counterstained: Bismarck brown & eosin, *in bulk*: borax carmine & counterstained: Azan (older embryos) | * |
| *Gallus gallus*  "chick" | Bellairs [21] | * | 4 - 10d  (cranial length) | whole mounts & serial sections | van Wijhe method (methylene blue), haematoxylin & eosin, or Masson's trichrome | * |
| *Gallus gallus* | Heyns [74] | 6 | 4 - 18d | serial sections | Ehrlichr's haematoxylin, and Bismarck brown & eosin | * |
| *Gallus gallus*  "*Gallus domesticus*" | Parker [58] | 10 | 4d - adult | sections | * | * |
| *Gallus gallus*  "Hühnchen" | Sonies [64] | 5 | 4 - 10d (6 - 26mm) | whole mounts & serial sections | victoria blue Van Wijhe method [111], *in toto:* haemalaun & counterstained: acid fuchsine & methylene blue | 16 µm - 20 µm |
| *Gallus gallus* | Vorster [69] | 6 | 4 - 18d | whole mounts & serial sections | eosin (youngest stages), Mayer's haemalum (younger embryos), Bismarck brown & eosin, Heidenhain's iron haematoxylin & Bismarck brown & eosin (older embryos), or azocarmine & azan (older embryos) | 10 µm & 12 µm |
| *Meleagris gallopavo* | Atalgin and Kürtül [33] | 4 | 9 - 12d | whole mounts | alcian blue & alizarin red [112] |  |
| *Meleagris gallopavo* | Maxwell [37] | 7 | 10 - 24d | whole mounts | alcian blue & alizarin red [112] |  |
| *Coturnix japonica* | Nakane and Tsudzuki [34] | 14 | 3 - 16d | whole mounts | alcian blue 8GX & alizarin red |  |
| *Coturnix japonica*  "*Coturnix coturnix japonica*" | Abd El-Hady [75] | 2 | St. 1 - 2 (35 - 49mm) | serial sections | *in toto:* borax-carmine & counterstained: picroindigo-carmine | 10 µm & 15 µm |
| *Anas platyrhynchos*  “*Anas* *boschas*” “duck” | de Beer and Barrington [46] | 14 | 132hr - 17d | whole mounts & serial sections | victoria blue Van Wijhe method [111,113], *in bulk*: borax-carmine & counterstained: picro-indigo-carmine, or Azan by Heidenhain | * |
| *Anas platyrhynchos*  "Ente" | Sonies [64] | 4 | 6 - 8d (11 - 14mm), 10d (22.5 & 23.5mm) | whole mounts & serial sections | victoria blue Van Wijhe method [111], *in toto*: Haemalaun & counterstained: acid fuchsine & methylene blue | 15 µm - 20 µm |
| *Spheniscus demersus* | Crompton [47] | 8 | 34 - 170mm  (tip to beak to cloacal opening) | serial sections | *in toto:* borax-carmine or Mayer's basic haemalum & counterstained: Azan, *in toto*: borax-carmine & counterstained: bleu de Lyon in Bismarck brown (in some cases additionally carbol fuchsine), *in toto*: basic haemalum & Bismarcks brown, and carbol fuchsine | * |
| *Phalacrocorax carbo* | Slabý [61] | 6 | 10 – 30mm | serial sections | haematoxylin & eosin | 8 µm - 12 µm |
| *Falco tinnunculus* "*Tinnunculus alaudarius*" | Suschkin [48] | 16 | St. I-XVI | serial sections | *in toto:* Hämocalcium (nach P. Mayer), or *in toto:* borax carmine & haematoxylin | 10 µm - 20 µm |
| *Melopsittacus undulatus* | de Kock [49] | 9 | 8 - 41mm (CRL) | serial sections | Heidenhain's iron haematoxylin, haemalum and Bismarck brown & erythrosin, or azocarmine, borax carmine & Azan | 10 µm, 12 µm, 15 µm |
| *Melopsittacus undulatus* | Lang [51] | 3 | St. A - C (2.2 - 5.5mm (head length) | serial sections | haemalum-eosin, Mallory, Azan by Heidenhain | 14 µm, 16 µm, 31? µm, 20 µm |
| *Euplectes orix*  "*Pyromelana orix orix*" | Engelbrecht [50] | 10 | St. I-X (18 - 57mm) (tip of beak to tip of tail) | serial sections | *in toto:* Mayer's basic haemalum & counterstained: Bismarck brown & eosin | 10 µm, 15 µm, 20 µm |
| *Melanosuchus niger* | Vieira *et al.* [72] | 6 | 10d - ? (St. 9 - 24) | whole mounts & serial sections | alcian blue & alizarin red, haematoxylin & eosin | 6 µm? |
| *Caretta caretta* | Kuratani [76] | 8 | St. I-VIII (6.8 - 23.4mm)  (carapace length) | whole mounts & serial sections | alcian blue & alizarin red; haematoxylin & eosin, or Azan | 10 µm - 15 µm |
| *Ptyodactylus hasselquistii* | El‐Toubi and Kamal [77,78] | 7 | 8 - 29d (15.2 - 26.5mm) (total body length) | serial sections | *in toto:* borax-carmine & counterstained: picroindigo-carmine | * |
| *Lacerta agilis* | Yaryhin and Werneburg [15] | 11 | 14 - 36d | whole mounts & serial sections | haematoxylin & eosin, and alcian blue | 7 µm - 10 µm |
| *Chalcides ocellatus* | El‐Toubi and Kamal [79,80] | 6 | 16.5 - 42mm  (total body length) | serial sections | *in toto:* borax-carmine & counterstained: picroindigo-carmine, or eosin & haematoxylin, and Mallory triple stain | * |

* no information available

**Additional references of Table S1**

111. Van Wijhe JW. A new Methode for demonstrating cartilaginous Mikroskeletons. Proc Sect Biol. 1902;47–53.

112. Dingerkus G, Uhler LD. Enzyme clearing of alcian blue stained whole small vertebrates for demonstration of cartilage. Stain Technol. 1977;52:229–32.

113. Van Wijhe JW. Frühe Entwicklungsstadien des Kopf- und Rumpfskeletts von *Acanthias vulgaris*. Bijdr tot Dierkd. 1922;22:271–98.
