# Supplementary material for: Evolution and development of the bird chondrocranium
Source: Front Zool. 2021 Apr 29;18:21. doi: 10.1186/s12983-021-00406-z (PMC8082637; doi:10.1186/s12983-021-00406-z)
Supplement: Supplementary file 2 — Additional file 2: Table S2. Terminology of the processes of the trabecular-polar region and their presumed homology [25, 47, 48, 50, 64]. [file 12983_2021_406_MOESM2_ESM.docx]

**Table S2** Terminology of the processes of the trabecular-polar region and their presumed homology [25,47,48,50,64].

| Species | Reference | Infrapolar process | Basitrabecular process |
| --- | --- | --- | --- |
| *Apteryx* sp. | Parker [57] | basipterygoid process |  |
| *Apteryx* sp. | de Beer [11] | * | * |
| *Dromaius novaehollandiae* | Lutz [28] | Processus basitrabecularis | Processus basipterygoideus |
| *Struthio* sp. | de Beer [11] | * | * |
| *Struthio* sp. | Brock [25] | * | * |
| *Struthio* sp. | Frank [65] | * | * |
| *Struthio camelus* | Lang [45] |  | Processus pterygoidei basisphenoidei |
| *Coturnix japonica* | Abd El-Hady [75] |  | quadratopolar commissure |
| *Gallus gallus* | Parker [58] | lingulae sphenoidales |  |
| *Gallus gallus* “Hühnchen” | Sonies [64] | * |  |
| *Gallus gallus* | Lang [51] | * |  |
| *Gallus gallus* | Vorster [69] | * |  |
| *Anas platyrhynchos* | Sonies [64] | * |  |
| *Anas platyrhynchos* | de Beer and Barrington [46] | * | * |
| *Columba* “Taube” | Filatoff [67] | trabecular part of Columella | Columella |
| *Columba livia domestica* | Lang [51] | Basitrabekel |  |
| *Columba livia* | El-Shikha [68] |  | quadratopolar commissure |
| *Streptopelia senegalensis* | Zaher and Riad [54] |  | quadratopolar commissure |
| *Spheniscus demersus* | Crompton [47] | * & infrapolar commissure** | * |
| *Phalacrocorax carbo* | Slabý [61] | * | * |
| *Falco tinnunculus* | Suschkin [48] | Processus basitrabecularis | Processus basipterygoideus |
| *Melopsittacus undulatus* | Lang [51] | * |  |
| *Melopsittacus undulatus* | de Kock [49] | * |  |
| *Euplectes orix* | Engelbrecht [50] | infracarotid commissure** | quadratopolar commissure |
| *Passer* “Sperling” | Sonies [64] | * | * |
| *Passer domesticus* | de Beer [11] | * |  |
| *Sturnus* “Star” | Sonies [64] | * | * |

* same terminology used

** the infrapolar process is defined here as a structure bounding the lateral carotid foramen without considering its formation
