# Supplementary material for: Evolution and development of the bird chondrocranium
Source: Front Zool. 2021 Apr 29;18:21. doi: 10.1186/s12983-021-00406-z (PMC8082637; doi:10.1186/s12983-021-00406-z)
Supplement: Supplementary file 3 — Additional file 3: Table S3. Selection of anatomical terms used for homologous chondrocranial characters in the bird chondrocranial literature. [file 12983_2021_406_MOESM3_ESM.docx]

**Table S3** Selection of anatomical terms used for homologous chondrocranial characters in the bird chondrocranial literature.

| acrochordal cartilage | mittlerer Balken [114], middle/median trabecula [60], prochordal plate [57], dorsum sellae [28,64], crista sellaris [73] |
| --- | --- |
| anterior orbital cartilage | preoptic root of orbital cartilage [46] |
| parachordals | Belegungsmasse/-platte [114], basal plate [60], investing mass [60], Umhüllungsmasse [48], Parachordalia [59], perichordal plate [47] |
| concha nasalis | upper turbinal [58] |
| fenestra ovalis | Foramen ovale [48,46,64], fenestra vestibuli [45] |
| fenestra basicranialis posterior | posterior basicranial fontanelle [56] |
| interorbital septum | perpendicular, vertical or middle ethmoidal plate [56,58], presphenoid [57] |
| metotic cartilage | paroccipital wing [51], paroccipital process [57], Occipitalflügel [48], processus subcapsularis [45] |
| maxilloturbinal | inferior turbinal [58] |
| nasal septum | internasal/ethmoidal cartilage [60], ethmo-vomerine plate [58], mesethmoid [57] |
| parietotectal cartilage | aliethmoid, aliseptal plates, and alinasal laminae [56] |
| pila antotica | lamina antotica [64] |
| planum antorbitale | pars plana [48,56,58], aliethmoid/ectoethmoid 5 [57], Praefrontale [53], anteorbital plate or prefrontal plate [45], lamina orbitonasalis [11] |
| planum supraseptale | Orbitosphenoidplatten/Orbitosphenoidea [48], orbitosphenoids [56,45], orbitosphenoid plates [57], lamina orbitonasalis [64], plana supraseptalia [25] |
| prenasal process | prenasal cartilage [57], fronto-nasal process [60], processus praenasalis [64] |
| tectum synoticum | Supraoccipitalplatte[48], superoccipital plate/lamina or exoccipital lamina [58], supraoccipital cartilage [97] |
| trabeculae | trabeculae cranii [60] |

**Additional reference for Table S3**

114. Rathke H. Entwickelungsgeschichte der Natter (*Coluber natrix*). Koenigsberg: Verlag der Gebrüder Bornträger; 1839.
