# Supplementary material for: Evolution and development of the bird chondrocranium
Source: Front Zool. 2021 Apr 29;18:21. doi: 10.1186/s12983-021-00406-z (PMC8082637; doi:10.1186/s12983-021-00406-z)
Supplement: Supplementary file 4 — Additional file 4: Table S4. Compilation of the original stages of species used for the analysis of the chondrification sequence. Continued in Table S5, Additional file 5. [file 12983_2021_406_MOESM4_ESM.docx]

**Table S4** Compilation of the original stages of species used for the analysis of the chondrification sequence. Continued in Table S5, Additional file 5.

|  | *Struthio camelus*  Parker [56] | *Struthio* sp.  Brock [25] | *Struthio* sp.  Frank [65] | *Struthio camelus*  Lang [45] | *Gallus gallus*  Huxley [60] | *Gallus gallus*  Parker [58] | *Gallus gallus*  Tonkoff [73] | *Gallus gallus*  Sonies [64] | *Gallus gallus*  Bellairs [21] | *Gallus gallus*  Voster [69] | *Gallus gallus*  Heyns [74] | *Coturnix japonica*  Nakane & Tsudzuki [34] | *Coturnix coturnix japonica*  Abd El Hady [75] | *Meleagris gallopavo*  Maxwell [37] |
| --- | --- | --- | --- | --- | --- | --- | --- | --- | --- | --- | --- | --- | --- | --- |
| acrochordal cartilage | A | St. 1 | 4mm |  |  | St. 1 | 10-11d | 5-5.5 mm | 4-5d | St. 1 | St. 1 | 3d | 35mm |  |
| parachordal cartilage | A | St. 1 | 5.4mm | St. A | 6d |  | 10-11d | 6 mm | 4-5d | St. 1 | St. 1 | 4d | 35mm |  |
| fenestra basicranialis posterior |  | ? | 0 | ? |  | St. 3 |  | 12 mm |  | St. 3 | St. 3 |  | ? |  |
| trabeculae |  | St. 1 | 5.4mm | St. A | 6d | St. 1 |  | 11 mm | 6ds | St. 1 | St. 2 | 5d | 35mm |  |
| trabecula communis | A |  | 8.4mm |  |  | St. 1 |  | 11-12 mm |  |  |  |  | 35mm |  |
| otic capsule | A | St. 2 | 9.2mm |  | 6d | St. 1 | 10-11d | 11-12 mm |  | St. 2 | St. 2 | 5d | 35mm | St. 33 |
| fenestra ovalis | A | ? | 12.4mm | St. A |  | St. 2 |  | 16 mm |  | St. 3 | St. 3 |  | 49mm |  |
| metotic cartilage |  | St. 3 | 12.4mm | St. A |  |  |  | 16.5-17 mm |  | St. 3 | St. 3 |  | 35mm |  |
| nasal septum | A | St. 2 | 12.3mm | St. A |  | St. 2 | 10-11d | 14-15 mm | 7d | St. 3 |  | 6d | 35mm | St. 33 |
| fenestration of nasal septum |  | ? |  | 0 |  | St. 4 | 10-11d | 20 mm | 8-10d | St. 4 | St. 4 |  | 40mm |  |
| prenasal process | A | St. 2 | 15.5mm | St. A |  | St. 2 | 10-11d | 14-15 mm |  | St. 3 | S. 3 | 7d | 49mm | St. 33 |
| planum antorbitale |  | St. 3 | 15.5mm | St. A |  | St. 2 |  | 20 mm | 7d |  | St. 3 |  | 43mm | St. 33 |
| parietotectal cartilage | B | St. 3 | 15.5mm | St. A |  | St. 2 |  | 14-15 mm | 5-7d | St. 3-4 |  |  | 35mm | St. 33 |
| nasal capsule |  | St. 4 | 19mm | St. A |  | St. 4 |  |  |  |  | St. 6 | 9d? | 49mm |  |
| cupola anterior |  |  | 21d |  |  |  | 10d? |  |  | St. 6 | St. 6 | 11d | 49mm | St. 33 |
| concha nasalis | B | St. 3 | 12.3mm | St. A |  |  |  | 17 mm |  |  | St. 5 | 7d? | 46mm |  |
| maxilloturbinal | B | St. 3 | 17.3mm |  |  |  |  | 20 mm |  | St. 5 | St. 4 | 7d? | 46mm |  |
| atrioturbinal | B | St. 4 | 21d |  |  |  |  |  |  | St. 5 |  | 7d? | 43mm |  |
| interorbital septum | A | St. 3 | 10.7mm | St. A |  | St. 2 | 10-11d | 16 mm | 8-10d | St. 3 | St. 3 | 7d? | 35mm | St. 33 |
| fenestration of interorbital septum |  | ? | ? | 0 |  | St. 4 | 10-11d | 20 mm |  | St. 6 | St. 6 | 9d? | ? | St. 33 |
| planum supraseptale |  | St. 2 | 12.3mm | St. A |  |  | 10-11d | 16 mm | 8d |  |  |  | ? |  |
| pila antotica |  | St. 2 | 5.4mm | St. A |  |  |  | 10-12 mm |  | St. 2 | St. 2 |  | 35mm |  |
| tectum synoticum | A |  | 21mm | St. A |  | St. 2 | 10-11d | 21 mm |  | St. 4 |  | 9d? | ? | St. 36 |
