# Supplementary material for: Evolution and development of the bird chondrocranium
Source: Front Zool. 2021 Apr 29;18:21. doi: 10.1186/s12983-021-00406-z (PMC8082637; doi:10.1186/s12983-021-00406-z)
Supplement: Supplementary file 5 — Additional file 5: Table S5. Compilation of the original stages of species used for analysis of the chondrification sequence. Continued from Table S4, Additional file 4. [file 12983_2021_406_MOESM5_ESM.docx]

**Table S5** Compilation of the original stages of species used for analysis of the chondrification sequence. Continued from Table S4, Additional file 4.

|  | *Meleagris gallopavo*  Atalgin and Kürtül [33] | *Anas platyrhynchos*  Sonies [64] | *Anas platyrhynchos*  de Beer and Barrington [46] | *Spheniscus demersus*  Crompton [47] | *Phalacrocorax carbo*  Slabý [61] | *Falco tinnunculus*  Suschkin [48] | *Melopsittacus undulatus*  Lang, [51] | *Melopsittacus undulatus*  de Kock [49] | *Euplectes orix*  Engelbrecht [50] | *Melanosuchus niger*  Vieira *et al*. [72] | *Caretta caretta*  Kuratani [76] | *Ptyodactylus hasselquistii*  El-Toubi and Kamal [77,78] | *Lacerta agilis*  Yaryhin and Werneburg [15] | *Chalcides ocellatus*  El-Toubi and Kamal [79,80] |
| --- | --- | --- | --- | --- | --- | --- | --- | --- | --- | --- | --- | --- | --- | --- |
| acrochordal cartilage |  | 9.5-10mm | St. 1 | St. 1 | 12.3mm | St. 1 | St. A | St. 2 | St. 1 | St. 10 | St. 1 | St. 1  (15.2mm) | St. 1 | St. 3  (23.3mm) |
| parachordal cartilage | 8d | 10mm | St. 1 | St. 1 | 10mm | St. 1 | St. A | St. 2 | St. 1 | St. 12 | St. 1 | St. 1  (15.2mm) | St. 1 | St. 1  (16.5mm) |
| fenestra basicranialis posterior |  | 13-14mm | St. 2 | 0 | 10mm | St. 3 | 0 | 0 | St. 2 | St. 14? | St. 3 | St. 3  (19.8mm) | St. 2 | St. 3  (23.3mm) |
| trabeculae | 9d | 10-10.5mm | St. 3 | St. 2 | 15mm | St. 2 | St. B | St. 2 | St. 3 | St. 9 | St. 1 | St. 1  (15.2mm) | St. 2 | St. 1  (16.5mm) |
| trabecula communis |  | 14mm | St. 7 | St. 2 | 15mm | St. 3 | St. B | St. 2 | St. 2 | St. 11 | St. 2 | St. 3  (19.8mm) | St. 2 | St. 3  (23.3mm) |
| otic capsule | 9d | 13-14mm | St. 1 | St. 1-3 | 15mm | St. 1 |  | St. 2-4 | St. 3 | St. 12 | St. 1 | St. 2  (16.8mm) | St. 3 | St. 2  (19mm) |
| fenestra ovalis |  | 15-16mm | St. 9 | St. 4-7 | 20mm | St. 3 | St. C | St. 4 | St. 4 |  | ? | St. 4  (21.3mm) | St. 7 | 42mm |
| metotic cartilage |  | 15-15.5mm | St. 8 | St. 1-2 | 15mm | St. 3 | St. B | St. 4 | St. 3 |  | 0 |  | 0 |  |
| nasal septum |  |  | St. 7 | St. 3 | 20mm? | St. 2 | St. B | St. 2 | St. 2 | St. 11 | St. 3 | St. 4  (21.3mm) | St. 4 | St. 3  (23.3mm) |
| fenestration of nasal septum | 12d? |  | St. 13 | St. 7 | ? | St. 5 | St. A* | St. 7 | St. 6 |  | ? | 38d  (37.8mm) | St. 10 | 42mm |
| prenasal process | 12d? |  | St. 7 | St. 2 | 12.3mm | St. 3 |  | St. 4 | St. 4 |  | 0 | 0 | 0 | 0 |
| planum antorbitale |  |  | St. 9 | St. 5 | 20mm | St. 2 | St. C | St. 4 | St. 3 | St. 12 | ? | St. 5  (22.6mm) | St. 5 | St. 5  (34.2mm) |
| parietotectal cartilage |  | 20mm | St. 8 | St. 5 | 20mm | St. 3 | St. C | St. 3 | St. 4 | St. 12 | St. 3 | St. 5  (22.6mm) | St. 7 | St. 4  (25.7mm) |
| nasal capsule |  |  | St. 14 | St. 6 | 30mm | St. 6 | St. C | St. 6 | St. 6 | St. 12 | St. 7? | St. 5  (22.6mm) | St. 9 | St. 5  (34.2mm) |
| cupola anterior |  |  | St. 14 | St. 8 | 30mm? | St. 6 | St. B* | St. 6 | St. 6 |  | St. 7? | St. 6  (23.8mm) | St. 10 | St. 5  (34.2mm) |
| concha nasalis |  |  | St. 9 | St. 6 | ? | ? |  | 0 | 0 | St. 12 | ? | St. 7  (26.5mm) |  | St. 5  (34.2mm) |
| maxilloturbinal |  |  | St. 13 | St. 7 | 30mm | St. 5 |  | St. 6 | St. 6 |  | ? |  |  |  |
| atrioturbinal |  |  | St. 14 | St. 8 | 30mm | St. 6 |  | St. 7 | St. 6 |  | ? |  |  |  |
| interorbital septum |  |  | St. 8 | St. 2 | 15mm | St. 2 | St. C | St. 4 | St. 3 | St. 12 | St. 2 | St. 4  (21.3mm) | St. 2 | St. 3  (23.3mm) |
| fenestration of interorbital septum |  |  | St. 13 | St. 7 | 30mm | St. 4 |  |  | St. 7 |  | ? | 0 | St. 8 | 42mm |
| planum supraseptale |  | 18.5-19mm |  | St. 6 | 23mm? | St. 6 | St. C | St. 6 |  | St. 12 | St. 3 | St. 4  (21.3mm) | St. 2 | St. 3  (23.3mm) |
| pila antotica |  | 14mm | St. 2 | St. 1 | 12.3mm | ? | St. B | St. 2 | St. 1-2 | St. 12? | St. 4 | St. 4  (21.3mm) | St. 2 | St. 5  (34.2mm) |
| tectum synoticum | 12d? | 19mm | St. 10 | St. 5-8 | 20mm | St. 5 | St. B* | St. 6 | St. 4 | St. 14 | St. 6 | St. 7  (26.5mm) | St. 6 | St. 5  (34.2mm) |
